# Insight into the Genetic Components of Community Genetics: QTL Mapping of Insect Association in a Fast-Growing Forest Tree

**DOI:** 10.1371/journal.pone.0079925

**Published:** 2013-11-19

**Authors:** Jennifer DeWoody, Maud Viger, Ferenc Lakatos, Katalin Tuba, Gail Taylor, Marinus J. M. Smulders

**Affiliations:** 1 Centre for Biological Sciences, Life Sciences, University of Southampton, Southampton, United Kingdom; 2 Institute of Silviculture and Forest Protection, University of West-Hungary, Sopron, Hungary; 3 Plant Research International, Wageningen UR Plant Breeding, Wageningen, The Netherlands; 4 Current address: USDA Forest Service, National Forest Genetics Lab, 2480 Carson Road, Placerville, California, United States of America; University of Calgary, Canada

## Abstract

Identifying genetic sequences underlying insect associations on forest trees will improve the understanding of community genetics on a broad scale. We tested for genomic regions associated with insects in hybrid poplar using quantitative trait loci (QTL) analyses conducted on data from a common garden experiment. The F_2_ offspring of a hybrid poplar (*Populus trichocarpa* x *P. deltoides*) cross were assessed for seven categories of insect leaf damage at two time points, June and August. Positive and negative correlations were detected among damage categories and between sampling times. For example, sap suckers on leaves in June were positively correlated with sap suckers on leaves (P<0.001) but negatively correlated with skeletonizer damage (P<0.01) in August. The seven forms of leaf damage were used as a proxy for seven functional groups of insect species. Significant variation in insect association occurred among the hybrid offspring, including transgressive segregation of susceptibility to damage. NMDS analyses revealed significant variation and modest broad-sense heritability in insect community structure among genets. QTL analyses identified 14 genomic regions across 9 linkage groups that correlated with insect association. We used three genomics tools to test for putative mechanisms underlying the QTL. First, shikimate-phenylpropanoid pathway genes co-located to 9 of the 13 QTL tested, consistent with the role of phenolic glycosides as defensive compounds. Second, two insect association QTL corresponded to genomic hotspots for leaf trait QTL as identified in previous studies, indicating that, in addition to biochemical attributes, leaf morphology may influence insect preference. Third, network analyses identified categories of gene models over-represented in QTL for certain damage types, providing direction for future functional studies. These results provide insight into the genetic components involved in insect community structure in a fast-growing forest tree.

## Introduction

Describing the genetic mechanisms underlying species interactions is a central aim of community genetics. This relatively new field of study expands the principles of population genetics to the associated species and larger ecosystem [1], [2], [3]. Since Whitham *et al*. [1] formalized the framework of the field numerous studies have examined the genetic basis of species interactions [4], [5], [6], [7], [8], [9], [10]. For example, in naturally occurring hybrid *Populus* systems, plant genotype has been shown to predict arthropod community structure [6], [9], to be related to the occurrence and abundance of invertebrate herbivores and their avian predators [5], and to influence the soil microbial community [11]. Further, these patterns of community association were found to be heritable and consistent over years, indicating that community stability may have a genetic component [12]. Evidence from common garden studies of evening primrose demonstrated the importance of genotype by environment interactions in community structure, with host genotype being significantly important in local micro-habitats [13].

Studies to identify possible mechanisms underlying community interactions often focus on major resistance genes or biochemical products of known function. In the natural *Populus* system, the role of condensed tannins has been well documented as influencing nutrient cycling and possibly community structure [14]. In addition, quantitative trait loci (QTL) have been identified for the important defense-related formylated phloroglucinols chemicals in *Eucalyptus globules* [15]. Alternatively, some studies have demonstrated that plant phenology may be critical to community interactions [16], [17], [18]. Leaves are the primary sites of interaction with herbaceous insects, and the life history traits, gross morphology, and defensive structures of plants may also play a significant role in the complex relationship between herbivores and hosts [19]. Such non-canonical mechanisms may underlie significant relationships where biochemical mechanisms have been ruled out.

The mechanisms influencing the community structure may be identified through quantitative genetic analyses, which do not rely on *a priori* understanding of causal traits. Using such a quantitative genetic approach to identify loci significantly correlated with community structure may reveal genetic mechanisms other than those predicted by secondary compounds or defensive traits. Additional insight into the genetic basis of community interactions may come by combining QTL and genomic data for the host species [20]. For example, mining gene models within QTL regions can narrow the search for candidate genes for future functional assays.

Forest trees provide a powerful system to examine the relationship between host genotype and phytophagous insects due to the long-lived nature of the host organism, relative frequency of insect occurrence on trees, and ease of assessing insect abundance. *Populus* species (cottonwoods, aspens, and poplars) frequently act as keystone species within their community, and are associated with a large number of insect, vertebrate, and fungal species [21]. Studies into the relationship between genetic variation in *Populus* host species and the diversity of the associated insect community have shed light on the complexities of community genetics [5], [6], [9], [12]. In the studies of *Populus fremontii*, *P. angustifolia* and their hybrids, significant correlations were identified between the genetic similarity of individual trees, the chemical properties of their leaves, and the structure of the insect communities on each individual [6]. These studies have focused on anonymous genetic differences (AFLP markers) between unrelated genetic individuals from natural stands, a system that does not provide insight into which genomic regions underlie the genetic variance. While insect associations on hybrid *Salix* have revealed genomic regions (quantitative trait loci, QTL) associated with insect damage in willow [22], to date no such study has been published for *Populus*.

In this study we combined traditional quantitative trait loci experiments with the genomic data available for *P. trichocarpa* to examine the genetic variance in insect community structure on hybrid poplar. Conducting a QTL study on a poplar pedigree provided the ability to investigate possible genetic mechanisms using the vast genomic resources available. The genomic sequence for *Populus trichocarpa*, a model tree [23], [24], was the first published for a woody species [25], and *Populus* species have been the focus of transcriptomic studies [26], [27], [28], [29], [30], functional genetic assays [31], [32], [33], in silico genomic studies [34], [35], [36], [37], and QTL and association genetic tests [38], [39], [40], [41], [42], [43].

**Figure 1:**
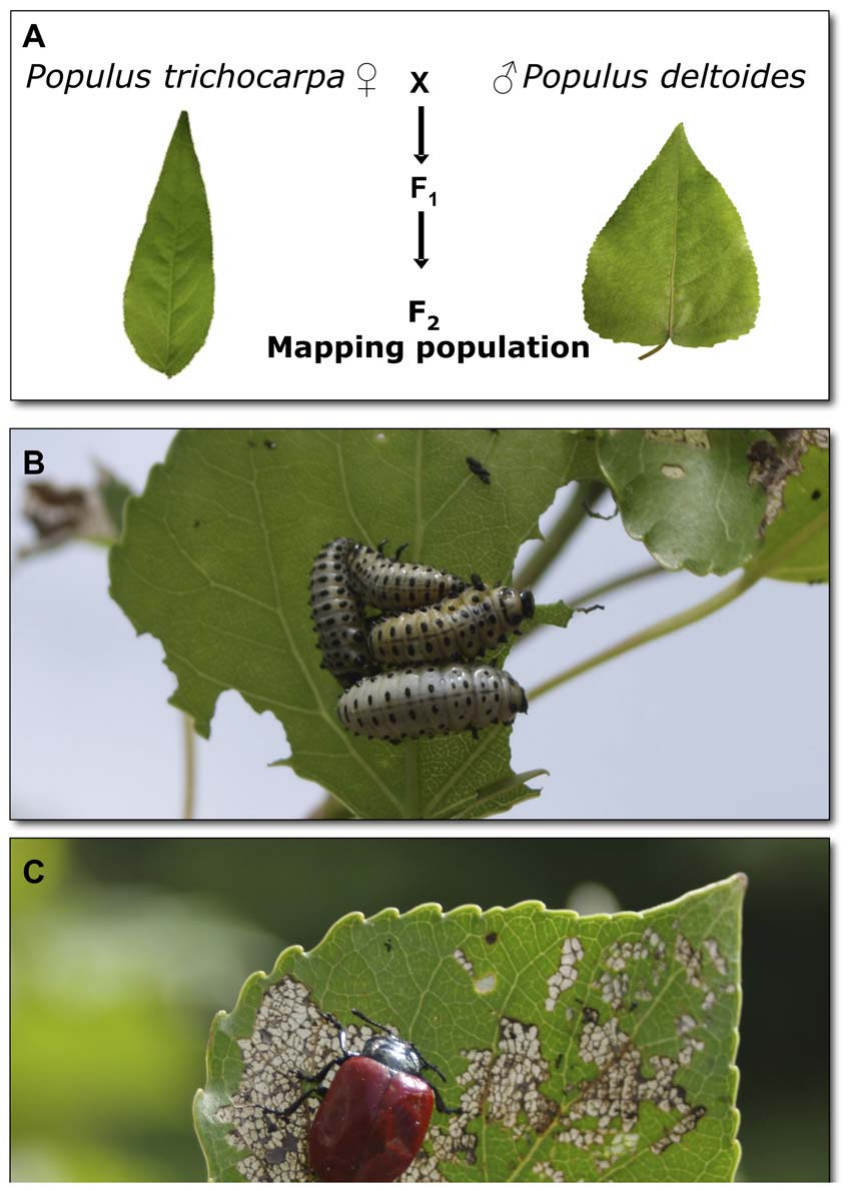
Insect association in a hybrid pedigree of *Populus trichocarpa* x *P. deltoides*. (A) Two F_1_ progeny were crossed to produce an F_2_ pedigree of full-sib trees that were propagated in a replicated common garden experiment. As an example of associated insect species, *Chrysomela populi* was a common chewing species observed on the hybrid poplar, shown in its (B) larval and (C) adult form.

We examined the correlation between genomic variation and insect association using quantitative trait loci (QTL) analysis. As the insects are difficult to detect, but the damage they cause can be assessed at any time, variation in insect association was classified into seven damage types (chewer, skeletonizer, leaf miner, gall damage, leaf rollers, and sap suckers on the leaf or stem), and quantified through visual inspection of leaf damage in an F_2_ pedigree of *P. trichocarpa* x *P*. *deltoides* (Fig. 1). After identifying QTLs, we combined them with genomic data by asking a series of questions. First, did QTL for insect association co-locate with shikimate-phenylpropanoid pathway genes (as identified by [44]), typically involved in plant defense? Second, did any QTL co-locate to genomic hot spots involved in leaf development? Third, were any gene families (defined through gene ontology categories) over-represented in the QTL for specific insect categories? Using genomic resources to examine the genes underlying the QTL for insect association provides insight into the possible mechanisms driving community genetics, and narrows the list of candidate genes for future studies. The results provided insights into possible genes or pathways underlying the community genetics of *Populus* systems.

## Materials and Methods

### Study system and field assessment

In North America, *Populus trichocarpa* (black cottonwood) and *P. deltoides* (eastern cottonwood) are widely distributed and can occur sympatrically [45]. *P. trichocarpa* (sect. *Tacamahaca*) and *P. deltoides* (sect. *Aigeiros*) are most easily distinguished by leaf size and shape, with the former displaying lanceolate leaves and the latter deltoid to cordate leaf bases [45]. The species also differ in leaf color and margin, branch and bud color, and floral characteristics [45]. Due to their interfertility and hybrid vigour when crossed, these species of *Populus* have been repeatedly chosen for hybrid analyses into the genetic basis of complex phenotypic traits [39], [46], [47].

Family 331 is a F_2_ hybrid pedigree of *Populus trichocarpa* x *P. deltoides*, consisting of the maternal grandparent *P. trichocarpa*, 93-968, crossed with the paternal grandparent *P. deltoides*, ILL-129, to produce hybrid F_1_ progeny (Fig. 1). Two of the F_1_ trees, the female 53-242 and male 53-246, were then crossed in two years to produce a full-sib hybrid pedigree (Family 331) [48], [49]. This study analyzed 189 genets (genotypes) of this pedigree, including the maternal grandparent (93-968), the two F_1_ parents (53-242 and 53-246) and 184 of the F_2_ progeny. One pure *P. deltoides* genet was analyzed to assess the variation in the paternal species, but the paternal grandparent was not available for assessment. In the spring of 2000, three replicates (ramets) of each genet were planted in a fully replicated randomized block design as part of an ongoing short rotation coppice experiment. The field work was undertaken at a closed Forest Research, Forestry Commission UK nursery site at Headley, Hampshire, U.K. (51°07' N, 0°50' W), with their permission, as previously described [50]. The trees were managed by Forest Research and were coppiced in the winter of 2009 prior to the start of the growing season, so this experiment assessed insect interactions on the first year of growth in the coppice cycle (summer 2009).

The genetic linkage map for the Family 331 pedigree was kindly provided by G. Tuskan (pers. comm.). The map was constructed from microsatellite (simple sequence repeats, SSR) and fully-informative amplified fragment length polymorphism (AFLP) markers using JoinMap v. 3.0 [51]. Specifically, 350 F_2_ progeny were genotyped for 91 microsatellite markers, and 165 F_2_ progeny were genotyped for 92 AFLP markers. The linkage map resolved 21 Linkage Groups defining a total map distance of 1,453.1 cM, with markers spaced an average of 8 cM across the map. Linkage maps in this F_2_ family were described elsewhere [42], [52]. They consistently indicated some levels of segregation distortion, and may include more than 19 linkage groups [42], which in part result from the use of dominant AFLP markers, which may increase the genotyping error. Although other linkage maps have been reported for this cross [53], [54], [55], our map increased the portion of the map aligned to the *P. trichocarpa* genome (v. 2.0). For this, the forward and reverse primer sequences for each microsatellite locus were aligned to the genomic sequence using BLASTN with a word length of 4. Alignments were conducted using linear interpolation over each marker pair via the tools provided on www.phytozome.net/poplar (accessed 3 May 2010), and revealed some putative inversions between the linkage map and physical sequences, consistent with observations in other pedigrees [42], [56] or possible genotyping error.

Interactions between insects and trees were quantified as different categories of leaf damage. Plants were scored for damage in June and August 2009. Each individual ramet score was the average of 30 leaves chosen at random from each plant. Damage was scored in 7 categories. Percentage of leaf area lost was scored for chewer, skeletonizer, miner and gall damage. The number of leaf rollers was counted, and the presence or absence of sap suckers on the leaf or stem (distinct categories) was scored. Measurements were made by three observers with the aid of printed guides depicting a scale of leaf damage (in percentages), photos of damage types for each class, and images of common phytophagous insect species. Prior to scoring and intermittently while in the field, the three observers scored the same tree and compared mean damage scores to maintain standardized measures while assessing the common garden. Finally, the observers changed starting locations for the August assessment in order to minimize the potential of influencing block effects by scoring the same plants twice.

### Data analysis

Due to the skewed nature of the data collected, raw measures were either arcsine transformed (for percentage measures) or square root transformed data (for count measures) prior to analysis. Transformed data were treated to the general linear model (GLM):
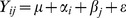
where *Y_ij_* is the phenotype of the *i*th genet in the *j*th block, α*_i_* is the genet (within individual) effect, β*_j_* is the block effect, and ε is the residual error. As there was no replication within blocks, this was a fully cross-factored model with no nesting. In addition, due to mortality of ramets in the field site, replication was reduced from the initial design so that approximately 30% of genets were represented by three replicates, 34% by two replicates, and 36% by a single replicate. Those traits found to have a significant block effect (June data for chewer, skeletonizer and leaf sucker; August data for chewer, skeletonizer, galls, leaf roller, and leaf sucker) were treated to a block correction by adding the difference of the block mean and the grand mean to each ramet score within each block. These block-corrected values were then treated to the same GLM to gain corrected estimates of within and among genet variance. Broad-sense heritability (*H* ^2^) was then estimated for each trait as:
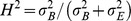
where 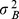 was estimated from the among-genet mean squares (MS_B_) and error mean squares (MS_E_) from the GLM (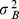  = (MS_B_ – MS_E_)/*r*, where *r* is the number of replicates) and 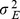 was estimated from the residual variance (ε) [50].

In order to assess phenotypic correlations between different categories of insects, Pearson’s correlation coefficient was calculated over genet means for all pairs of damage categories and scoring month. Block-corrected data were used when appropriate. The correlation coefficients and 2-tailed measures of significance were calculated in SPSS v. 17.0 (IBM Corporation, Somers, New York).

To assess variation in insect community structure across genets, a non-metric multidimensional scaling (NMDS) ordination procedure was applied. Specifically, we were interested in quantifying differences in the composition of the insect community associated with each tree, rather than differences in each damage category. The NMDS procedure reduces multivariate (here, community) data to a smaller number of orthogonal ordination axes, and has been repeatedly applied to similar questions [5], [9], [11], [12]. The NMDS ordination was based on the Bray-Curtis distance matrix [57] calculated from the damage scores for all 13 damage types by month categories. Ordination scores for each ramet were extracted for *k* = 2 axes, and variation among genets was examined using a distance matrix-based analysis of variance based on the linear model described for the univariate analyses above. Permutations of raw data values were used to estimate pseudo-F statistics for the terms in the linear model. The analysis provided estimates of variance (mean squares) that were then used to estimate the broad-sense heritability of community structure following the manner described for individual damage levels. All analyses were conducted using the multiMDS and adonis functions provided in the R package vegan (The R Project for Statistical Computing).

The mean scores for each genet were treated to a quantitative trait loci (QTL) analysis. QTL were identified using Grid-QTL [58], a Grid portal analysis system based on the algorithms used by QTLExpress [59]. QTL were identified using the ONE-QTL interval mapping method, which we consider a conservative approach compared to a TWO-QTL model, given the low-density nature of the linkage map employed and the modest number of F_2_ progeny in this study. Significance was determined through 1000 randomizations of all markers along a linkage group, with permutation results used to estimate the critical value (*F*-ratio) for each trait-chromosome combination [59], [60]. A QTL was considered significant when the test statistic at an interval was greater than the critical value defined by permutation tests for that experiment. The permutation approach can provide a more robust estimate of a critical value than using an *a priori* value for likelihood ratio or LOD scores [60]. For each significant QTL identified, its position (cM) on the linkage group was defined as the interval with the greatest test statistic and the 95% confidence interval for the QTL location was defined as all intervals on the chromosome with a test statistic greater than the critical value. In addition, the percent variance in the trait explained was recorded. In order to describe differences in parental alleles, the paternal (*P. deltoides*) and maternal (*P. trichocarpa*) effects were calculated following [61]. As Family 331 is an outcrossed pedigree, it is assumed that each grandparent (P_0_, pure parental species) was heterozygous at each locus so that the effects of four alleles are considered in each estimate. This approach is justified by the high level of heterozygosity revealed by resequencing of multiple *Populus* genotypes [25], [62].

We then undertook a three-step approach to identify potential mechanisms underlying each QTL. First, the physical location of shikimate-phenylpropanoid pathway genes (as described by [44]) on the *Populus* genome were plotted in order to identify co-location of insect association QTL and possible biochemical defences. Second, a test for co-location between insect association and leaf trait QTL was conducted. A total of 105 QTL for leaf traits identified in Family 331 were collected from studies in the UK [40], [50], [63], including the same site studied here [50], and in Italy [64] (Table S2 in Supporting Information). Significant overlap of QTL from multiple environments provides evidence of genetic (not only genetic by environment) variance. Eight categories of leaf traits were included: leaf area, leaf extension rate, leaf length, leaf width, leaf length:width ratio, leaf mass, absolute expansion rate, and specific leaf area. For studies testing the effects of abiotic treatments [40], [63], only QTL identified for control (ambient) traits were included. After the QTL identified for insect associations were added to the list, the distribution of QTL were assessed for non-random alignment across the Family 331 genetic map using a (5 cM) sliding-window approach as previously described [39]. Significance was determined from 2000 permutations of the QTL locations across all linkage groups, identifying regions of the genome having a greater density of QTL than expected at random. All co-location analyses and QTL plotting were conducted in qtlplots, a package developed for use in the R statistical environment by Nathaniel Street and available from the author (nathaniel.street@plantphys.umu.se).

Third, a bioinformatics analysis of functional categories of genes in QTL regions was used to identify genes or genetic pathways for further analysis. We aligned the position and 95% confidence interval of each significant QTL with the *Populus trichocarpa* physical map (v. 2.0) using regional distance ratios defined by the two anchored microsatellite markers closest to each QTL. This allowed us to extract an approximate bp location of each QTL and 95% confidence interval in the genomic sequence. All gene models, whether well annotated or not, within each confidence interval were identified and extracted using the BioMart tool at www.phytozome.net/poplar (accessed October 2010). Gene ontology categories for three independent classifications (biological processes, molecular function, and cellular component) were extracted for each gene model [65]. To identify over-represented categories of gene models, the frequency of gene ontology categories within each insect category (e.g. skeletonizer damage in June) was compared to the distribution of gene ontology categories for the *P. trichocarpa* genome as a whole. Tests were conducted using singular enrichment analysis (SEA), with adjustments for multiple tests made using Benjamini-Hochberg false discovery rate (FDR) for p = 0.05. All analyses were conducted in agriGO v. 1.2, a web-based analysis service that provides gene model GO classification and background level analyses [66].

## Results

Significant variation in levels of damage was observed for the majority of insect categories scored among the full-sib genets, indicating that the insect community is associated with poplar genets in a non-random manner. Damage levels varied across guilds, and the majority of trees showed little damage in several categories (Table 1). In both June and August, three categories of damage showed significant differences in damage levels among genets: chewers, skeletonizers and sap suckers on leaves (Table 2). The *P. trichocarpa* parent and F_1_ progeny displayed lower damage levels than many F_2_ genets (Table 1). While the *P. deltoides* parent was not available, a half-sib genotype was included in the common garden and experienced lower damage than many of the F_2_ progeny (data not presented). Thus, the distribution of damage levels in the F_2_ progeny is consistent with transgressive segregation of susceptibility to insect damage.

**Table 1:** Average levels of leaf damage and broad-sense heritability (*H* ^2^) observed for seven categories of insect herbivory on an F_2_ pedigree of hybrid poplar.

	*P. trichocarpa*	F_1_ (242)	F_1_ (246)	F_2_ ± 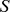	*H* ^2^
**Category**					
*June data*					
Chewer*	0.744	0.456	1.74	2.38±0.18	0.164
Skeletonizer*	1.46	2.84	4.28	4.03±0.30	0.284
Leaf miner*	0.022	0.0	0.033	0.009±0.001	0.000§
Gall*	0.0	0.0	0.0	0.005±0.0004	0.000§
Leaf roller†	0.0	0.0	0.0	0.0006±0.00004	0.027
Sap sucker, leaf‡	0.256	0.078	0.044	0.074±0.005	0.229
Sap sucker, stem‡	0.0	0.0	0.0	0.002±0.0001	0.000§
*August data*					
Chewer*	1.2	2.48	2.17	5.37±0.40	0.137
Skeletonizer*	1.56	1.69	1.72	3.09±0.23	0.152
Leaf miner*	0.0	0.533	0.344	0.077±0.006	0.098
Gall*	0.0	0.033	0.0	0.004±0.0003	0.221
Leaf roller†	0.0	0.011	0.0	0.003±0.0002	0.139
Sap sucker, leaf‡	0.0	0.033	0.156	0.117±0.009	0.312
Sap sucker, stem‡	No damage				n/a

* Percent leaf area damaged.

† Count.

‡ Proportion leaves scored with damage present.

§ Negative calculation truncated to zero.

**Table 2:** Significant genetic variation (factor Genet) in levels of insect association (damage) among progeny of a hybrid pedigree.

	June				August		
Category	Factor	MS	*F*		Factor	MS	*F*
Chewer	Genet	34.18	*F*_188,187_ * = *1.44**		Genet	23.22	*F*_186,175_ * = *1.40*
	Block	102.2	*F*_2,187_ * = *4.29*		Block	71.68	*F*_2,175_ * = *4.33*
	Error	23.82			Error	16.56	
Skeletonizer	Genet	20.95	*F*_188,187_ * = *2.16***		Genet	12.38	*F*_186,175_ * = *1.74***
	Block	347.1	*F*_2,187_ * = *35.76***		Block	492.9	*F*_2,175_ * = *69.3***
	Error	9.71			Error	7.11	
Leaf miner	Genet	0.2801	*F*_188,187_ * = *0.70		Genet	2.559	*F*_186,175_ * = *1.21
	Block	0.0447	*F*_2,187_ * = *0.11		Block	0.1801	*F*_2,175_ * = *0.09
	Error	0.3981			Error	2.113	
Gall	Genet	0.1719	*F*_188,187_ * = *0.50		Genet	0.1436	*F*_186,175_ * = *1.48**
	Block	0.8466	*F*_2,187_ * = *2.46		Block	0.4469	*F*_2,175_ * = *4.60*
	Error	0.3438			Error	0.0971	
Leaf roller	Genet	0.0005	*F*_188,187_ * = *1.08		Genet	0.002	*F*_186,175_ * = *1.23
	Block	0.00002	*F*_2,187_ * = *0.05		Block	0.007	*F*_2,175_ * = *3.43*
	Error	0.0005			Error	0.002	
Sap sucker, leaf	Genet	131.4	*F*_188,187_ * = *1.48**		Genet	189.5	*F*_186,175_ * = *1.95***
	Block	856.9	*F*_2,187_ * = *9.65***		Block	463	*F*_2,175_ * = *4.78**
	Error	88.83			Error	96.92	
Sap sucker, stem	Genet	6.28	*F*_188,187_ * = *0.71		Genet	No damage	n/a
	Block	21.38	*F*_2,187_ * = *2.42		Block		
	Error	8.843			Error		

* *P*<0.05; **P<0.01, ***P<0.001.

**Figure 2:**
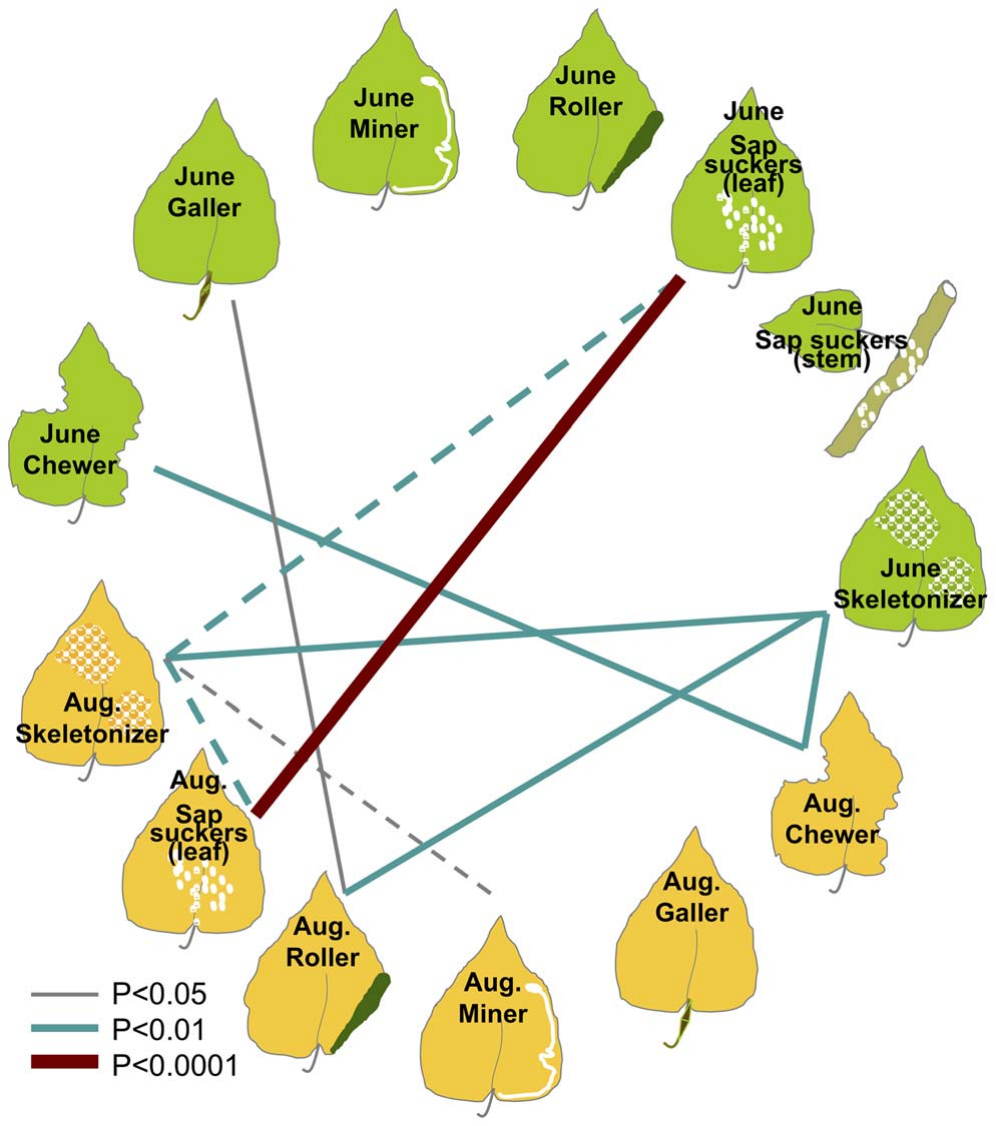
Significant correlation in damage levels on hybrid poplar observed among insect guilds and months. Solid lines represent positive correlations; dashed lines depict negative correlations. Line thickness and color corresponds to the significance of the correlation.

Correlations between damage levels varied among insect categories and sampling times. Nine pairs of damage types were significantly correlated (Fig. 2, Table S1). Three damage types were significantly positively correlated between early (June) and late (August) summer: chewer (*r = *0.213, d.f. * = *181, *P = *0.004), sap suckers on leaves (*r = *0.996, d.f. * = *181, *P* < 0.0001), and skeletonizers (*r = *0.249, d.f. * = *181, *P = *0.001). Damage by skeletonizers in June was positively correlated with August levels of chewer damage (*r = *0.232, d.f. * = *181, *P = *0.002) and leaf roller presence (*r = *0.194, d.f. * = *181, *P = *0.008). Similarly, the presence of leaf galls in June was positively correlated with the occurrence of leaf rollers in August (*r = *0.156, d.f. * = *181, *P = *0.035). Skeletonizer damage in August was negatively correlated with the presence of sap suckers on leaves in June (*r = *−0.219, d.f. * = *181, *P = *0.003), indicating these species may avoid previously damaged leaves or be deterred by ant mutualists (which were observed but not quantified during the season). Patterns of damage in August revealed a negative correlation between the damage by skeletonizers and both the number of leaf miners (*r = *−0.157, d.f. * = *181, *P = *0.034) and the presence of sap suckers on leaves (*r = *−0.218, d.f. * = *181, *P = *0.003), indicating that skeletonizers may deter other phytophagous insects, or that the insect community may be stratified across the tree canopy.

**Figure 3:**
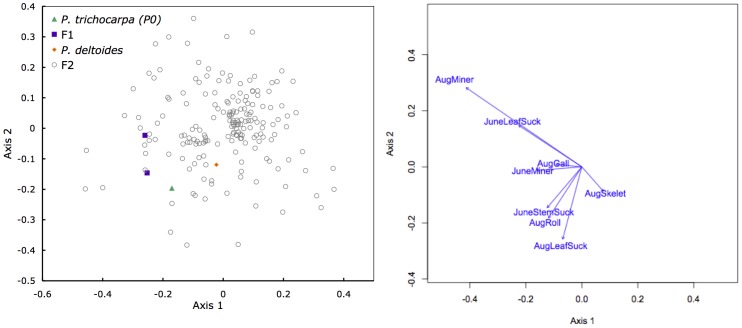
Non-metric multidimensional scaling (NMDS) ordination of the community structure among hybrid poplar genets (A) and the insect damage categories assessed (B). Variance among genets indicated the community structure is moderately heritable among the F_2_ progeny (*H* ^2^ *= *0.137).

Examination of insect community structure using NMDS (Fig. 3) revealed significant variation among genets (*F*_186, 175_ *= *1.476, *P = *0.001). The variance among genets corresponded to a broad-sense heritability of *H* ^2^ *= *0.137. These values indicate genetic factors influence community structure as a whole, not just individual insect guild host choice, in hybrid poplar.

A total of 14 QTL were identified for seven of the season-trait combinations assessed (Table 3, Fig. S1). No significant QTL were associated with leaf rollers, gall damage, or sap suckers on stems. A smaller number of QTL were identified for the June data than August data, consistent with the higher levels of damage observed later in the season. For the June data, one QTL was identified for each of three damage categories: chewer, skeletonizer, and leaf miner, each on a different linkage group (Table 3). These QTL explained between 3.0 and 7.7% of the phenotypic variance observed in these traits.

**Table 3:** Quantitative trait loci (QTL) identified for categories of insect damage assessed on an F_2_ pedigree of hybrid poplar in June and August. The position and 95% confidence interval are provided in cM.

Category	LG	Position (cM)	95% CI	*P*-value	% Variance	Maternal effect	Paternal effect	LOD score
*June*								
Chewer	Vb	4	0–19	0.008	3.33	–0.115 (0.348)	–0.985 (0.342)	1.763
Miner	I	9	0–24	0.014	7.69	0.006 (0.003)	0.008 (0.003)	2.259
Skeletonizer	XIV	0	0–28	0.032	3.02	–0.444 (0.290)	–0.709 (0.290)	1.639
*August*								
Chewer	III	37	29–46	<0.001	9.27	–1.085 (0.286)	–0.625 (0.275)	4.212
Chewer	IV	59	45–85	0.012	4.55	–0.569 (0.341)	0.829 (0.303)	2.244
Chewer	Va	76	62–86	0.015	4.92	0.807 (0.299)	0.608 (0.296)	2.392
Chewer	XVII	54	35–69	0.007	5.41	1.313 (0.395)	0.469 (0.379)	2.593
Leaf Miner	Va	19	0–41	0.020	4.14	0.031 (0.017)	0.045 (0.016)	2.083
Leaf Miner	VIIIa	27	12–27	0.015	3.65	0.032 (0.016)	–0.038 (0.016)	1.878
Leaf Miner	XVII	50	33–70	0.005	5.73	–0.035 (0.022)	–0.054 (0.021)	1.181
Sap Sucker, Leaf	I	74	32–125	0.041	4.81	–0.035 (0.015)	0.032 (0.015)	2.368
Sap Sucker, Leaf	VI	144	134–144	0.006	6.73	–0.012 (0.009)	–0.035 (0.009)	3.158
Sap Sucker, Leaf	XII	17	0–24	0.037	2.88	–0.022 (0.010)	0.014 (0.009)	1.622
Skeletonizer	III	14	0–31	0.005	5.81	–0.468 (0.131)	–0.065 (0.153)	2.757

Significance (*P*-value) was determined from 1000 chromosome-wide permutations. Standard errors in parentheses.

For the August data, 11 QTL were identified for four damage categories: chewer, skeletonizer, leaf miner, and sap suckers on leaves (Table 3). Individual QTL identified for August damage levels explained between 2.9 and 9.3% of the phenotypic variance observed in these traits. In total, the QTL observed for each trait explained 24% of the phenotypic variance in chewer damage, 5.8% in skeletonizer damage, 13% for leaf miner damage, and 14% for the presence of sap suckers on leaves.

A three-step approach using genomic resources was taken to identify possible mechanisms or candidate genes for future study. First, we identified QTL containing shikimate-phenylpropanoid pathway genes within the 95% confidence interval. These genes represent potential biochemical defence pathways that may serve as selective agents against phytophagous insects. The number of shikimate-phenylpropanoid genes within a QTL ranged from 0 to 4 (Table 4). Given the total length of the linkage map (1453.1), and the number of genes examined (74), we expected one gene every 20 cM under an even distribution. A Chi-squared test of the number of shikimate-phenylpropanoid genes observed per QTL revealed the distribution to be no different than this random prediction (*χ* ^2^ *= *12.96, d.f. = 12, *P* = 0.371).

**Table 4.Identification:** of potential mechanisms underlying QTL for insect association in hybrid poplar using genomic resources: co-location of genes involved in phenolic glycoside (PG) production, leaf morphology QTL “hot spots”, and analysis of gene ontologies.

						Number of GO Categories Over-represented		
Category	LG	QTL (cM)	# PG genes	Hot spot?	N Gene Models	Biological Processes	Cellular Component	Molecular Function
*June* *								
Leaf miners	I	0 – 24	4		1174	59	15	58
Skeletonizers	XIV	0 – 28	1	Yes	1850	87	29	75
*August*								
Chewers	III	29 – 46	0		301	114	32	66
Chewers	IV	45 – 85	1		465			
Chewers	Va	62 – 86	1		396			
Chewers	XVII	35 – 69	2		455			
Miners	Va	0 – 41	1		1933	85	37	69
Miners	VIIIa	12 – 27	0		292			
Miners	XVII	33 – 70	2		505			
Sap Suckers - leaves	I	32 – 125	2		1910	78	33	69
Sap Suckers – leaves	VI	134 – 144	0		118			
Sap Suckers - leaves	XII	0 – 24	1	Yes	414			
Skeletonizer	III	0 – 31	0		666	9	17	22

* Microsatellite primers failed to resolve the placement of LG Vb, prohibiting analysis of the June chewer QTL.

Second, a catalogue of QTL for eight leaf traits from three previous studies identified a total of 106 QTL (Table S2 in Supporting Information). The number of QTL per trait ranged between five (absolute expansion rate) to 33 (leaf area). When the insect association QTL were added to the list of leaf trait QTL, a total of 126 QTL were assessed for random distribution across the genome. Tests for co-location between insect damage and leaf trait QTL identified two genetic ‘hotspots’ for leaf morphology alone and two ‘hotspots’ containing QTL for leaf and insect traits. An insect QTL was adjacent to the leaf QTL hotspot on LG VIIIa, but no insect QTL was proximate to the hotspot on LG IX (data not presented). The leaf+insect hotspots occurred on linkage groups XII and XIV (Fig. S2). The hotspot on LG XII included the QTL for sap suckers on leaves in August, and the hotspot on LG XIV included the QTL for skeletonizers in June, indicating that these regions may include both leaf development and insect interaction loci, or that the insect community may respond to variation in leaf development controlled by genes in this chromosomal region.

Third, bioinformatics analyses identified between 119 and 1933 gene models (open reading frames identified either from expressed sequence tags or predictive algorithms in the genome annotation) within individual QTL regions (Table 4). The large numbers are partly due to the unsaturated nature of the genetic linkage map for the F_2_ pedigree, which resulted in relatively large confidence intervals on some linkage groups. Analysis of the distribution of gene ontology (GO) classifications for each gene model revealed a non-random pattern of gene function within insect categories. All but two damage category/GO class combinations displayed at least nine over-represented GO categories, with a maximum of 114 in the chewer QTL for August damage (Table 4, Table S3 in Supporting Information).

## Discussion

### Community structure revealed by correlations among insect guilds

Our assessment revealed significant variation in insect association among hybrid poplar genotypes in both early and late summer. Levels of leaf damage were correlated among time points: Genets damaged by chewers, skeletonizers or sap suckers on leaves in June were more likely to display higher levels of the same damage in August. This pattern was likely due in part to genetic basis of insect preference when choosing host plants, consistent with the heritable patterns of insect richness on *Populus fremontii*, *P. angustifolia* and naturally occurring hybrids [9], [12]. Significant correlations of insect abundance has been reported among years in *Salix* [67]. Correlation within damage types may also reflect the potential sedentary nature of some insect species or life stages (e.g. aphids) and will likely vary among years and locations. Insect damage can be episodic, and significant damage by one species may affect the pattern and influence of other insect herbivores.

In addition to individual phytophagous categories, the insect community structure, as quantified through NMDS analysis, was moderately heritable in this population, providing evidence of the extended phenotype of hybrid poplar trees. The broad-sense heritability estimated for the F_2_ progeny indicates genotype explained 13% of the variation in community structure among trees, a smaller percentage than was estimated for tri-trophic interactions among *P. angustifolia* and back-cross hybrids (*H* ^2^ *= *0.70, [5]), soil microbial community mass among *P. angustifolia* X *P. fremontii* F_1_ hybrids (*H* ^2^ *= *0.23) and soil microbial composition among *P.angustifolia* individuals (*H* ^2^ *= *0.70, [11]). The previous studies focused on the natural hybrid zone between *P. angustifolia* and *P. fremontii* in the southwest region of North America, sampling unrelated trees. The hybrid classes represented independent hybridizations between multiple parental genotypes. In our study, the F_2_ samples were full-sibs, representing a pedigree produced by the hybridization of one *P. deltoides* and one *P. trichocarpa* trees. The lower heritability values likely reflect the reduced amount of genetic variance available in the F_2_ population compared with wild-collected trees. Despite the caveat, these findings demonstrate that phytophagous insects respond to genetic differences even among related host plants, narrowing the search for the genetic mechanism of selection in trophic interactions.

### Quantitative trait loci for insect associations in hybrid poplar

Despite the economic and ecological importance of this genus, this is the first study to report QTL for insect association in *Populus*. We identified 14 regions of the hybrid *Populus* genome significantly associated with the feeding of four insect guilds (chewers, leaf miners, sapsuckers on leaves, and skeletonizers). These genomic regions are spread across nine linkage groups, indicating the insect community responds to wide ranges of genetic variation on host plants. Given the modest number of *F*_2_ progeny assessed for each trait (fewer than 200), the number of QTL identified likely underestimated the true number involved in these plant-insect interactions (biased towards QTL of large effect), yet overestimated the effect size of each QTL due to the Beavis effect [68], [69]. Nonetheless, our results are consistent with a QTL analysis for insect damage in hybrid *Salix* (willow), another member of the Salicaceae. In *Salix*, the number and position of QTL varied by site, with differences attributed in part to the composition of the local insect community [22]. In addition, QTL for different traits did not frequently overlap, and the few instances where QTL co-located were considered possible locations of defensive genes or gene complexes [22]. In *Populus*, QTL for different damage categories only co-located in one instance, with QTL for chewer and leaf miner damage in August occurring on LG XVII. The 95% confidence intervals for these QTL occupy a large portion of the linkage group, and the region is not robustly aligned to the physical map, but two shikimate-phenylpropanoid pathway genes, chorismate mutase (CM) and phenylalanine ammonia-lyase (PAL), are adjacent. Both genes are involved in the PAL-dependent phenolic glycoside pathway [44].

Insect levels [70] and leaf characteristics [67] vary throughout the growing season in tree populations, so this work assessed damage in early and late summer. QTL for three categories (chewer, miner, and skeletonizer) at the two time points located to different linkage groups, reflecting the dynamic relationship between leaf development and insect host choice through the season, or facultative and indirect defence mechanisms within plants [9], [71], [72].

The study site used for this work presents a significant caveat for the interpretation and transfer of these findings. The poplar genotypes presented novel host plants to the native insect community of southern England as the two parental species of the F_2_ cross are native to North America, where natural hybridization occurs but is infrequent [45]. Genotype by environment interactions may be most important over large geographic areas, and the role of host genotypic variation may be limited to a local scale. Examination of genotypic and environmental variation in arthropod abundance on *Oenothera biennis* showed that environmental variation drove species richness across diverse habitats, but that plant genotype explained a greater portion of the variance within microhabitats [13]. Thus, while the QTL identified here correspond to the community structure of insects in this non-native plantation of hybrid poplar, other genetic mechanisms will likely be involved in the extended phenotype of either *P. trichocarpa* or *P. deltoides* in their native range. Such a caveat should not nullify this study, for three reasons. First, as the first attempt to identify the quantitative variation underlying community genetics in a *Populus*, this study provides a proof of concept. We found significant heritability of insect association, although the level may be higher in the species’ native ranges. Given our ability to identify QTL for association with non-native insect species, it is likely that the same number or even more significant QTL would be identified for co-evolving insect species within the trees’ native ranges. Further, the poplar pedigree examined here has a wealth of genetic and genomic tools available, and has been the focus of numerous physiological studies in varying environments [40], [42], [55], [73]. Other hybrid poplars (including those using *P. deltoides* germplasm) are regularly used as stock in plantations across Europe, meaning these findings may transfer directly to ongoing studies and biomass breeding purposes. Second, examining native insects on a non-native host may provide insight into the genetic basis of novel species interactions. By examining the genetic basis of host choice in non-native tree species, these findings may better reflect the mechanism of host switching or invasion rather than the mechanism of adaptation in a natural stand. Repeating this study in different environment will be necessary to determine the robustness of these QTL, in particular any genotype by environment interactions that may influence the extended phenotype of these trees. Third, many of the F_2_ trees displayed community structure or individual insect association levels transgressive to those on the F_1_ parents and pure species. These observations are consistent with studies showing natural *Populus* hybrid zones to be a center of biodiversity [74], [75], [76], and further support the management and protection of hybrid complexes in conservation efforts.

### Possible mechanisms underlying QTL

**Figure 4:**
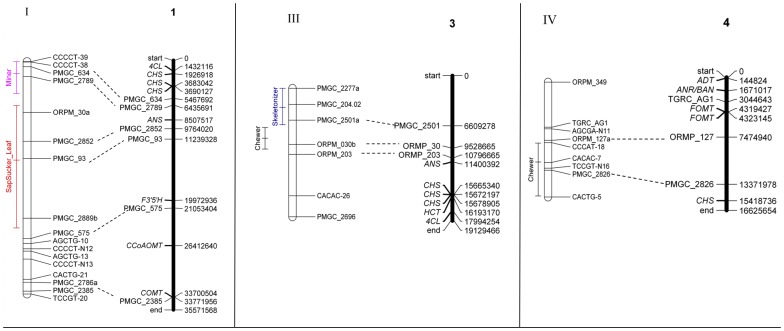
Location and 95% confidence intervals of QTL for insect association (quantified as leaf damage) in hybrid poplar. Open bars represent linkage groups for the F_2_ hybrid linkage map. Solid bars represent the physical map of *Populus trichocarpa*, which also depicts genes phenolic glycoside production as described in Tsai *et al*. (2006), in *italics*. *4CL*  =  4-Coumarate-CoA ligase; *ADT*  =  Arogenate dehydratase; *ANR/BAN*  =  Anthocyanidin reductase; *ANS*  =  Anthocyanidin synthase; *C3H*  =  Courmarate 3-hydroxylase; *CCoAOMT*  =  Caffeoyl-CoA *O*-methyltransferase; *CHS*  =  Chalcone synthase; *CM*  =  Chorismate mutase; *COMT*  =  Caffeic acid *O*-methyltransferase; *DFR*  =  Dihydroflavonol 4-reductase; *F3’5’H*  =  Flavonoid 3’,5’-hydroxylase; *FOMT*  =  Flavonoid *O*-methyltransferase; *HCT*  =  Hydroxycinnamoyl-CoA quinate/shikimate hydroxycinnamoyltransferase; *ICS*  =  Isochorismate synthase; *LAR*  =  Leucoanthocyanidin reductase; *PAL*  =  Phenylalanine ammonia-lyase. The two maps were aligned using microsatellite loci (dashed lines).

We harnessed the genomic resources available for *P. trichocarpa* to search for possible mechanisms underlying each QTL identified. Our three-step approach provides insight for future studies based on candidate gene sequence and functional analyses. First, nine of the 13 QTL examined against the *P. trichocarpa* physical map contained at least one of the shikimate-phenylpropanoid pathway genes described by [44]. The shikimate-phenylpropanoid pathway produces three families of secondary metabolites involved in plant defense or growth: phenolic glycosides, hydroxycinnamate derivatives, and condensed tannins [44]. The defensive role of phenolic glycosides has been described in detail in *P. tremuloides*, providing evidence of varying susceptibility among phytophagous insects, a possible mechanism of defense activity [77], and of genetic variance in glycoside levels among clones [78]. The abundance of the various compounds varies among tissues and developmental stage within the plant [44]. For example, the chalcone synthase gene family is greatly diversified in *Populus*, and members have been shown to be highly upregulated in response to wounding, indicating these genes may serve a defensive role [44]. Out of 16 shikimate-phenylpropanoid pathway genes to co-locate to insect association QTL, six were classified in the CHS family (Fig. 4), consistent with these genes affecting insect host choice. It may be possible that such insects respond to the phenolic characteristics of a host plant not to avoid compounds, but to identify appropriate food for their own defences. Leaf beetles in the subtribe Chrysomelina (Fig. 1), for instance, have developed strategies to use host plant glucosides in their own defence chemistry [79]. These genes are promising candidates for additional sequence diversity and functional assays. An alternative avenue for future research is to assess the levels of these components directly in leaves that are also scored for types and level of insect damage, enabling a direct correlation analysis while bypassing genotype by environment interactions [20].

Second, we examined the co-location of insect association and leaf morphology QTL to ask whether morphological and not strictly biochemical factors may affect insect host choice. Two QTL for insect association co-located to genomic hot-spots for leaf morphology. Both of these genomic regions also contain one gene involved in the shikimate-phenylpropanoid pathway, but the feeding guilds involved may respond to leaf morphology or phenology. A third QTL hotspot contained only leaf morphology QTL, with a QTL for leaf miner association in August adjacent (LG VIIIa). Insect association may reflect leaf morphology or biochemistry other than defensive compounds. Total leaf nitrogen content (indicative of photosynthetic potential) and leaf toughness may influence the level and diversity of phytophagous insects on a tree [67]. These factors change throughout the growing season, and vary with abiotic environmental conditions, meaning they may underlie key genotype by environment interactions in community structure. Correlation between insect association and leaf morphology may provide additional insight into links between plant development and insect preference in this *F*_2_ pedigree. Alternatively, the genomic hotspots containing both classes of QTL may be due to genetic linkage of independent causative genes or the pleiotropic effects of a single locus.

Finally, we tested for the over-representation of gene ontology categories within the QTL regions identified for each damage type. These inferences would be improved by increasing the density of the linkage map used in the analyses, which would be expected to reduce the confidence intervals of significant QTL. Nonetheless, similar GO classes were observed to be over-represented in QTL for the different damage types, indicating similar functional components may underlie the genetic basis of insect association. For instance, bioinformatics analyses identified gene models related to extracellular glutamate-gated ion channel activity to be overrepresented in QTL regions related to chewer abundance. These glutamate receptors (GLRs) are a class of ligand-gated, non-selective cation channels found in animals and plants [80]. While the role of mammalian glutamate receptors in neural signal transduction is well established, the complex roles of GLRs in plants are still being resolved. Recent studies indicate GLRs are involved in root morphogenesis [81], [82], Ca^++^ influx relating to stomatal movements [83], NO production in response to a fungal secretion [84], and abscisic acid synthesis and signalling [85]. Interestingly, GLRs are also involved in plant morphology and jasmonic acid signalling, including the production of defensins [86], consistent with their co-locating to QTL for chewer damage in hybrid *Populus*.

## Conclusions

By quantifying the direct interaction between phytophagous insects and hybrid poplar in a common garden experiment, we identified 14 QTL for insect association, revealing genomic regions putatively involved in the genetic components of community structure. Multivariate analyses revealed the community structure to vary among genotypes, consistent with a moderately heritable community trait (broad-sense heritability  =  0.13). Using the genomic resources available for *P. trichocarpa*, we identified possible mechanisms underlying the QTL, including shikimate-phenylpropanoid pathway genes, genomic hot-spots for leaf morphology, and over-represented gene ontology categories. Together, these genes help to narrow the list of candidate genes for future functional and sequence-based studies aiming to identify the mechanisms of community structure in this ecologically and economically important forest tree.

## Supporting Information

Table S1 **Pearson’s correlation coefficients among leaf damage categories assessed in early (June) and late (August) summer in hybrid poplar**
(PDF)

Table S2 **Leaf morphology QTL from three previous studies of hybrid poplar used for co-location analyses.**
(PDF)

Table S3 **Complete list of GO classifications over-represented in QTL for insect association identified in hybrid poplar.**
(PDF)

Figure S1 **Test statistics across each chromosome, calculated as** ***F*** **- ratios, were used to identify significant QTL for each month/damage category.** Each horizontal axis represents the length of one linkage group from the F_2_ linkage map. The vertical axis indicates the magnitude of the test statistic. Solid lines represent the observed *F-*ratio, and dashed lines represent the 5% critical value calculated from 1000 chromosome-wide permutations.
(PDF)

Figure S2 **QTL for insect damage (red) co-locate to QTL hotspots (gold) for leaf traits (green) on two linkage groups in a hybrid poplar pedigree (** ***P. trichocarpa*** **x** ***P. deltoides*** **,** ***T*** **x** ***D*** **).** (A) The presence of sap suckers (primarily aphids) on leaves co-locates with QTL for leaf size on LG XII. (B) The frequency of skeletonizers early in the summer co-locates with QTL for leaf size and growth rate on LG XIV.
(TIFF)
