# Supplementary material for: Insight into the Genetic Components of Community Genetics: QTL Mapping of Insect Association in a Fast-Growing Forest Tree
Source: PLoS One. 2013 Nov 19;8(11):e79925. doi: 10.1371/journal.pone.0079925 (PMC3833894; doi:10.1371/journal.pone.0079925)
Supplement: Table S1 — Pearson’s correlation coefficients among leaf damage categories assessed in early (June) and late (August) summer in hybrid poplar (PDF) [file pone.0079925.s001.pdf]

Table S1. Pearson's correlation among leaf damage categories assessed in early (June) and late (August) summer in hybrid poplar.

	Galler June	Leaf Miner June	Leaf Roller June	Sap Sucker Leaf June	Sap Sucker Stem June	Skelet-onizer June	Chewer Aug	Galler Aug	Leaf Miner Aug	Leaf Roller Aug	Sap Sucker Leaf Aug	Skelet-onizer Aug
Chewer_June	-0.005	-0.065	-0.011	-0.057	0.039	0.093	0.213**	-0.047	-0.061	-0.003	-0.058	0.033
Galler_June		-0.012	-0.017	-0.024	-0.018	0.012	-0.018	-0.024	0.056	0.156*	-0.023	-0.027
LeafMiner_June			-0.025	0.1	-0.043	0.029	-0.063	-0.063	0.091	-0.027	0.101	0.064
LeafRoller_June				0.035	0.051	0.059	-0.063	-0.026	-0.027	-0.065	0.038	0.117
SapSuckerLeaf_June					0.091	-0.085	0.042	-0.071	0.063	0.028	0.996***	-0.219**
SapSuckerStem_June						0.06	0.056	-0.035	-0.048	0.096	0.089	0.112
Skeletonizer_June							0.232**	0.091	0.006	0.194**	-0.047	0.249**
Chewer_Aug								-0.032	-0.076	0.122	0.044	-0.002
Galler_Aug									-0.055	0.085	-0.07	0.108
LeafMiner_Aug										-0.003	0.071	-0.157*
LeafRoller_Aug											0.036	0.015
SapSuckerLeaf_Aug												-0.218**

* P<0.05, **P<0.01, ***P<0.0001
