# Supplementary material for: Insight into the Genetic Components of Community Genetics: QTL Mapping of Insect Association in a Fast-Growing Forest Tree
Source: PLoS One. 2013 Nov 19;8(11):e79925. doi: 10.1371/journal.pone.0079925 (PMC3833894; doi:10.1371/journal.pone.0079925)
Supplement: Table S2 — Leaf morphology QTL from three previous studies of hybrid poplar used for co-location analyses. (PDF) [file pone.0079925.s002.pdf]

Table S2. Three previous studies identified 105 QTL for 8 leaf traits in a hybrid pedigree of poplar (POP1).

Leaf Trait	QTL Name	Reference	N
Leaf area	leaf_area_SRC1	Rae et al., 2004	4
	leaf_area_SRC2	Rae et al., 2004	4
	leaf_area_mature_aCO2	Rae et al., 2006	10
	leaf_area_young_aCO2	Rae et al., 2006	7
	leaf_area_leaf_Italy	Rodriguez-Acosta, 2006	2
	leaf_area_leaf_UK	Rodriguez-Acosta, 2006	6
Leaf extension rate	leaf_extension_SRC	Rae et al., 2004	4
	leaf_extension_aCO2	Rae et al., 2006	4
	leaf_extension_droughtcontrol	Street et al., 2006	1
Leaf length	leaf_length_leaf_Italy	Rodriguez-Acosta, 2006	2
	leaf_length_leaf_UK	Rodriguez-Acosta, 2006	6
Leaf width	leaf_width_leaf_Italy	Rodriguez-Acosta, 2006	1
	leaf_width_leaf_UK	Rodriguez-Acosta, 2006	7
	leaf_width_droughtcontrol16d	Street et al., 2006	2

**Leaf Trait**	**QTL Name**	**Reference**	**N**
	leaf_width_droughtcontrol9d	Street et al., 2006	1
Length:width ratio	leaf_width:length_aCO2	Rae et al., 2006	6
	leaf_length:width_leaf_Italy	Rodriguez-Acosta, 2006	4
	leaf_length:width_leaf_UK	Rodriguez-Acosta, 2006	5
	leaf_length:width_droughtcontrol	Street et al., 2006	2
Leaf mass	dry_weight_leaf_Italy	Rodriguez-Acosta, 2006	2
	dry_weight_leaf_UK	Rodriguez-Acosta, 2006	6
Absolute expansion rate	absolute_expansion_aCO2	Rae et al., 2006	2
	absolute_expansion_droughtcontrol	Street et al., 2006	2
Specific leaf area	SLA_SRC	Rae et al., 2004	6
	SLA_aCO2	Rae et al., 2006	2
	SLA_leaf_Italy	Rodriguez-Acosta, 2006	2
	SLA_leaf_UK	Rodriguez-Acosta, 2006	5
