# Supplementary material for: Insight into the Genetic Components of Community Genetics: QTL Mapping of Insect Association in a Fast-Growing Forest Tree
Source: PLoS One. 2013 Nov 19;8(11):e79925. doi: 10.1371/journal.pone.0079925 (PMC3833894; doi:10.1371/journal.pone.0079925)
Supplement: Table S3 — Complete list of GO classifications over-represented in QTL for insect association identified in hybrid poplar. (PDF) [file pone.0079925.s003.pdf]

Table S3. GO classification over-represented in QTL for insect assication identified in hybrid poplar.
GO ontologies classified into three categories: C=cellular components, F=molecular function, P=biological processes.
FDR = p-value following Benjamini-Hochberg adjustment for mulitiple tests for a false discovery rate of 0.05.

Trait	GO term	Ontology	Description	N in QTL	N in genome	p	FDR
ChewerAug	GO:0005623	C	cell	266	7109	3.10E-11	6.50E-10
ChewerAug	GO:0044464	C	cell part	266	7109	3.10E-11	6.50E-10
ChewerAug	GO:0005622	C	intracellular	170	4246	4.00E-10	5.60E-09
ChewerAug	GO:0043231	C	intracellular membrane-bounded organelle	93	2042	6.30E-09	6.70E-08
ChewerAug	GO:0043227	C	membrane-bounded organelle	93	2055	8.50E-09	7.10E-08
ChewerAug	GO:0044424	C	intracellular part	140	3536	1.60E-08	1.10E-07
ChewerAug	GO:0005634	C	nucleus	74	1626	1.70E-07	7.90E-07
ChewerAug	GO:0043226	C	organelle	113	2821	1.50E-07	7.90E-07
ChewerAug	GO:0043229	C	intracellular organelle	113	2821	1.50E-07	7.90E-07
ChewerAug	GO:0016020	C	membrane	109	3219	0.00019	0.00069
ChewerAug	GO:0033177	C	proton-transporting two-sector ATPase complex, proton-transporting domain	6	37	0.0002	0.00069
ChewerAug	GO:0044425	C	membrane part	60	1551	0.00018	0.00069
ChewerAug	GO:0030288	C	outer membrane-bounded periplasmic space	7	56	0.00031	0.001
ChewerAug	GO:0030313	C	cell envelope	7	59	0.00044	0.0011
ChewerAug	GO:0042597	C	periplasmic space	7	57	0.00035	0.0011
ChewerAug	GO:0044462	C	external encapsulating structure part	7	58	0.00039	0.0011
ChewerAug	GO:0016469	C	proton-transporting two-sector ATPase complex	8	80	0.00056	0.0014
ChewerAug	GO:0043234	C	protein complex	39	965	0.00093	0.0022
ChewerAug	GO:0032991	C	macromolecular complex	55	1499	0.001	0.0023
ChewerAug	GO:0016021	C	integral to membrane	42	1082	0.0013	0.0028
ChewerAug	GO:0005789	C	endoplasmic reticulum membrane	7	79	0.0025	0.0047
ChewerAug	GO:0042175	C	nuclear envelope-endoplasmic reticulum network	7	79	0.0025	0.0047
ChewerAug	GO:0044432	C	endoplasmic reticulum part	7	81	0.0028	0.005
ChewerAug	GO:0071212	C	subsynaptic reticulum	7	81	0.0028	0.005
ChewerAug	GO:0005783	C	endoplasmic reticulum	10	153	0.0033	0.0055
ChewerAug	GO:0044444	C	cytoplasmic part	40	1085	0.004	0.0064
ChewerAug	GO:0030312	C	external encapsulating structure	12	223	0.0065	0.01
ChewerAug	GO:0000151	C	ubiquitin ligase complex	8	122	0.0079	0.011
ChewerAug	GO:0031224	C	intrinsic to membrane	44	1270	0.0076	0.011
ChewerAug	GO:0031975	C	envelope	11	204	0.0088	0.012
ChewerAug	GO:0005737	C	cytoplasm	47	1393	0.0094	0.013
ChewerAug	GO:0005694	C	chromosome	8	139	0.016	0.022
ChewerAug	GO:0003824	F	catalytic activity	416	12409	8.90E-10	1.30E-07
ChewerAug	GO:0003676	F	nucleic acid binding	143	3583	7.60E-09	5.30E-07
ChewerAug	GO:0003677	F	DNA binding	86	2000	2.20E-07	1.00E-05
ChewerAug	GO:0005488	F	binding	386	12383	1.20E-06	4.10E-05
ChewerAug	GO:0016787	F	hydrolase activity	135	3729	2.40E-06	6.80E-05
ChewerAug	GO:0016740	F	transferase activity	160	4741	1.50E-05	0.00034
ChewerAug	GO:0000166	F	nucleotide binding	158	5094	0.00058	0.0037
ChewerAug	GO:0004175	F	endopeptidase activity	23	429	0.00025	0.0037
ChewerAug	GO:0004970	F	ionotropic glutamate receptor activity	7	65	0.00079	0.0037
ChewerAug	GO:0005083	F	small GTPase regulator activity	9	96	0.00042	0.0037
ChewerAug	GO:0005216	F	ion channel activity	11	145	0.00063	0.0037
ChewerAug	GO:0005230	F	extracellular ligand-gated ion channel activity	7	65	0.00079	0.0037
ChewerAug	GO:0005231	F	excitatory extracellular ligand-gated ion channel activity	7	65	0.00079	0.0037
ChewerAug	GO:0005234	F	extracellular-glutamate-gated ion channel activity	7	65	0.00079	0.0037
ChewerAug	GO:0005524	F	ATP binding	139	4427	0.0007	0.0037
ChewerAug	GO:0008066	F	glutamate receptor activity	7	65	0.00079	0.0037
ChewerAug	GO:0008233	F	peptidase activity	31	699	0.00068	0.0037

Trait	GO term	Ontology	Description	N in QTL	N in genome	p	FDR
ChewerAug	GO:0015267	F	channel activity	11	147	0.00071	0.0037
ChewerAug	GO:0015276	F	ligand-gated ion channel activity	7	65	0.00079	0.0037
ChewerAug	GO:0016772	F	transferase activity, transferring phosphorus-containing groups	105	3097	0.00023	0.0037
ChewerAug	GO:0016788	F	hydrolase activity, acting on ester bonds	35	830	0.0008	0.0037
ChewerAug	GO:0022803	F	passive transmembrane transporter activity	11	147	0.00071	0.0037
ChewerAug	GO:0022834	F	ligand-gated channel activity	7	65	0.00079	0.0037
ChewerAug	GO:0022838	F	substrate-specific channel activity	11	145	0.00063	0.0037
ChewerAug	GO:0030528	F	transcription regulator activity	54	1374	0.00025	0.0037
ChewerAug	GO:0030695	F	GTPase regulator activity	9	99	0.00053	0.0037
ChewerAug	GO:0032553	F	ribonucleotide binding	146	4698	0.0008	0.0037
ChewerAug	GO:0032555	F	purine ribonucleotide binding	146	4698	0.0008	0.0037
ChewerAug	GO:0032559	F	adenyl ribonucleotide binding	139	4429	0.00071	0.0037
ChewerAug	GO:0070011	F	peptidase activity, acting on L-amino acid peptides	31	676	0.0004	0.0037
ChewerAug	GO:0060589	F	nucleoside-triphosphatase regulator activity	9	112	0.0013	0.0058
ChewerAug	GO:0003700	F	transcription factor activity	36	891	0.0014	0.0062
ChewerAug	GO:0004252	F	serine-type endopeptidase activity	11	162	0.0016	0.0066
ChewerAug	GO:0017076	F	purine nucleotide binding	150	4945	0.0017	0.0068
ChewerAug	GO:0005215	F	transporter activity	50	1378	0.002	0.0082
ChewerAug	GO:0001882	F	nucleoside binding	141	4666	0.0024	0.0087
ChewerAug	GO:0001883	F	purine nucleoside binding	141	4664	0.0024	0.0087
ChewerAug	GO:0016301	F	kinase activity	83	2541	0.0023	0.0087
ChewerAug	GO:0030554	F	adenyl nucleotide binding	141	4664	0.0024	0.0087
ChewerAug	GO:0004842	F	ubiquitin-protein ligase activity	8	104	0.0031	0.01
ChewerAug	GO:0008270	F	zinc ion binding	43	1165	0.0029	0.01
ChewerAug	GO:0022836	F	gated channel activity	8	104	0.0031	0.01
ChewerAug	GO:0016790	F	thiolester hydrolase activity	7	87	0.0042	0.014
ChewerAug	GO:0016773	F	phosphotransferase activity, alcohol group as acceptor	79	2473	0.0047	0.015
ChewerAug	GO:0016779	F	nucleotidyltransferase activity	22	507	0.0047	0.015
ChewerAug	GO:0016746	F	transferase activity, transferring acyl groups	18	393	0.0057	0.017
ChewerAug	GO:0016616	F	oxidoreductase activity, acting on the CH-OH group of donors, NAD or NADP as accepto	10	167	0.0061	0.018
ChewerAug	GO:0004672	F	protein kinase activity	73	2301	0.0072	0.02
ChewerAug	GO:0008237	F	metallopeptidase activity	8	121	0.0076	0.02
ChewerAug	GO:0015078	F	hydrogen ion transmembrane transporter activity	7	96	0.0072	0.02
ChewerAug	GO:0016747	F	transferase activity, transferring acyl groups other than amino-acyl groups	17	371	0.007	0.02
ChewerAug	GO:0030234	F	enzyme regulator activity	15	311	0.007	0.02
ChewerAug	GO:0003964	F	RNA-directed DNA polymerase activity	14	294	0.0099	0.026
ChewerAug	GO:0004221	F	ubiquitin thiolesterase activity	6	80	0.011	0.028
ChewerAug	GO:0004091	F	carboxylesterase activity	11	212	0.012	0.029
ChewerAug	GO:0004806	F	triglyceride lipase activity	5	59	0.012	0.029
ChewerAug	GO:0005096	F	GTPase activator activity	5	59	0.012	0.029
ChewerAug	GO:0034061	F	DNA polymerase activity	15	329	0.011	0.029
ChewerAug	GO:0022857	F	transmembrane transporter activity	31	861	0.013	0.031
ChewerAug	GO:0016874	F	ligase activity	19	464	0.014	0.033
ChewerAug	GO:0016614	F	oxidoreductase activity, acting on CH-OH group of donors	10	192	0.015	0.035
ChewerAug	GO:0043565	F	sequence-specific DNA binding	23	606	0.017	0.039
ChewerAug	GO:0008047	F	enzyme activator activity	5	66	0.019	0.042
ChewerAug	GO:0016853	F	isomerase activity	10	200	0.02	0.043
ChewerAug	GO:0019787	F	small conjugating protein ligase activity	10	201	0.02	0.043
ChewerAug	GO:0016298	F	lipase activity	5	69	0.022	0.047
ChewerAug	GO:0044238	P	primary metabolic process	365	9502	3.40E-15	6.20E-13
ChewerAug	GO:0008152	P	metabolic process	432	12037	3.00E-13	2.80E-11
ChewerAug	GO:0043170	P	macromolecule metabolic process	289	7539	6.70E-13	4.20E-11

Trait	GO term	Ontology	Description	N in QTL	N in genome	p	FDR
ChewerAug	GO:0044237	P	cellular metabolic process	315	8506	3.80E-12	1.80E-10
ChewerAug	GO:0009987	P	cellular process	376	10572	9.90E-12	3.70E-10
ChewerAug	GO:0044260	P	cellular macromolecule metabolic process	256	6824	4.80E-11	1.50E-09
ChewerAug	GO:0006139	P	nucleobase, nucleoside, nucleotide and nucleic acid metabolic process	133	3290	1.10E-08	2.80E-07
ChewerAug	GO:0006807	P	nitrogen compound metabolic process	147	3784	2.40E-08	5.30E-07
ChewerAug	GO:0019538	P	protein metabolic process	169	4499	2.60E-08	5.30E-07
ChewerAug	GO:0009058	P	biosynthetic process	159	4271	9.50E-08	1.50E-06
ChewerAug	GO:0009059	P	macromolecule biosynthetic process	124	3136	8.90E-08	1.50E-06
ChewerAug	GO:0034645	P	cellular macromolecule biosynthetic process	124	3130	8.10E-08	1.50E-06
ChewerAug	GO:0009056	P	catabolic process	55	1073	1.50E-07	2.10E-06
ChewerAug	GO:0044249	P	cellular biosynthetic process	149	4007	2.20E-07	2.90E-06
ChewerAug	GO:0044267	P	cellular protein metabolic process	139	3849	2.00E-06	2.50E-05
ChewerAug	GO:0009057	P	macromolecule catabolic process	48	976	2.50E-06	2.90E-05
ChewerAug	GO:0010467	P	gene expression	106	2807	4.10E-06	4.50E-05
ChewerAug	GO:0006350	P	transcription	74	1864	1.80E-05	0.00018
ChewerAug	GO:0010468	P	regulation of gene expression	70	1734	1.70E-05	0.00018
ChewerAug	GO:0060255	P	regulation of macromolecule metabolic process	73	1837	2.00E-05	0.00018
ChewerAug	GO:0006508	P	proteolysis	39	799	2.20E-05	0.0002
ChewerAug	GO:0044248	P	cellular catabolic process	23	367	2.50E-05	0.00021
ChewerAug	GO:0009889	P	regulation of biosynthetic process	68	1720	4.10E-05	0.00027
ChewerAug	GO:0010556	P	regulation of macromolecule biosynthetic process	68	1720	4.10E-05	0.00027
ChewerAug	GO:0019219	P	regulation of nucleobase, nucleoside, nucleotide and nucleic acid metabolic process	68	1717	3.90E-05	0.00027
ChewerAug	GO:0031326	P	regulation of cellular biosynthetic process	68	1720	4.10E-05	0.00027
ChewerAug	GO:0045449	P	regulation of transcription	68	1713	3.70E-05	0.00027
ChewerAug	GO:0065007	P	biological regulation	95	2601	3.70E-05	0.00027
ChewerAug	GO:0080090	P	regulation of primary metabolic process	71	1821	4.40E-05	0.00028
ChewerAug	GO:0019222	P	regulation of metabolic process	73	1898	5.30E-05	0.00031
ChewerAug	GO:0043412	P	macromolecule modification	102	2866	5.30E-05	0.00031
ChewerAug	GO:0050789	P	regulation of biological process	91	2490	5.00E-05	0.00031
ChewerAug	GO:0031323	P	regulation of cellular metabolic process	68	1743	6.00E-05	0.00034
ChewerAug	GO:0051171	P	regulation of nitrogen compound metabolic process	68	1755	7.30E-05	0.0004
ChewerAug	GO:0006464	P	protein modification process	99	2799	8.20E-05	0.00044
ChewerAug	GO:0051179	P	localization	73	1942	0.0001	0.00054
ChewerAug	GO:0006259	P	DNA metabolic process	33	688	0.00012	0.0006
ChewerAug	GO:0050794	P	regulation of cellular process	84	2336	0.00015	0.00072
ChewerAug	GO:0006091	P	generation of precursor metabolites and energy	17	264	0.00019	0.00088
ChewerAug	GO:0044265	P	cellular macromolecule catabolic process	19	314	0.00019	0.00088
ChewerAug	GO:0015985	P	energy coupled proton transport, down electrochemical gradient	8	70	0.00022	0.00099
ChewerAug	GO:0015986	P	ATP synthesis coupled proton transport	8	70	0.00022	0.00099
ChewerAug	GO:0055085	P	transmembrane transport	13	175	0.00026	0.0011
ChewerAug	GO:0006793	P	phosphorus metabolic process	86	2462	0.0003	0.0012
ChewerAug	GO:0006796	P	phosphate metabolic process	86	2462	0.0003	0.0012
ChewerAug	GO:0006810	P	transport	70	1911	0.00028	0.0012
ChewerAug	GO:0051234	P	establishment of localization	70	1911	0.00028	0.0012
ChewerAug	GO:0034220	P	ion transmembrane transport	8	74	0.00033	0.0013
ChewerAug	GO:0006119	P	oxidative phosphorylation	9	97	0.00045	0.0017
ChewerAug	GO:0016310	P	phosphorylation	83	2409	0.00055	0.0021
ChewerAug	GO:0006260	P	DNA replication	20	382	0.00081	0.0029
ChewerAug	GO:0043687	P	post-translational protein modification	85	2529	0.00097	0.0035
ChewerAug	GO:0007265	P	Ras protein signal transduction	7	69	0.0011	0.0038
ChewerAug	GO:0046578	P	regulation of Ras protein signal transduction	7	69	0.0011	0.0038
ChewerAug	GO:0051056	P	regulation of small GTPase mediated signal transduction	7	69	0.0011	0.0038

Trait	GO term	Ontology	Description	N in QTL	N in genome	p	FDR
ChewerAug	GO:0006511	P	ubiquitin-dependent protein catabolic process	12	191	0.0019	0.0055
ChewerAug	GO:0006544	P	glycine metabolic process	5	38	0.0018	0.0055
ChewerAug	GO:0006818	P	hydrogen transport	8	95	0.0017	0.0055
ChewerAug	GO:0015992	P	proton transport	8	95	0.0017	0.0055
ChewerAug	GO:0019941	P	modification-dependent protein catabolic process	12	191	0.0019	0.0055
ChewerAug	GO:0043632	P	modification-dependent macromolecule catabolic process	12	191	0.0019	0.0055
ChewerAug	GO:0044257	P	cellular protein catabolic process	12	191	0.0019	0.0055
ChewerAug	GO:0051603	P	proteolysis involved in cellular protein catabolic process	12	191	0.0019	0.0055
ChewerAug	GO:0055086	P	nucleobase, nucleoside and nucleotide metabolic process	18	349	0.0017	0.0055
ChewerAug	GO:0009966	P	regulation of signal transduction	7	76	0.002	0.0056
ChewerAug	GO:0010646	P	regulation of cell communication	7	76	0.002	0.0056
ChewerAug	GO:0007264	P	small GTPase mediated signal transduction	7	78	0.0023	0.0064
ChewerAug	GO:0016567	P	protein ubiquitination	8	100	0.0024	0.0065
ChewerAug	GO:0009069	P	serine family amino acid metabolic process	5	41	0.0025	0.0068
ChewerAug	GO:0032446	P	protein modification by small protein conjugation	8	102	0.0027	0.0071
ChewerAug	GO:0070647	P	protein modification by small protein conjugation or removal	8	102	0.0027	0.0071
ChewerAug	GO:0044106	P	cellular amine metabolic process	17	345	0.0035	0.009
ChewerAug	GO:0006753	P	nucleoside phosphate metabolic process	15	298	0.0048	0.012
ChewerAug	GO:0009117	P	nucleotide metabolic process	15	298	0.0048	0.012
ChewerAug	GO:0006468	P	protein amino acid phosphorylation	73	2267	0.0052	0.013
ChewerAug	GO:0006519	P	cellular amino acid and derivative metabolic process	17	365	0.006	0.015
ChewerAug	GO:0006066	P	alcohol metabolic process	12	224	0.0068	0.016
ChewerAug	GO:0009308	P	amine metabolic process	18	404	0.0075	0.018
ChewerAug	GO:0051276	P	chromosome organization	7	101	0.0094	0.022
ChewerAug	GO:0006278	P	RNA-dependent DNA replication	14	294	0.0099	0.023
ChewerAug	GO:0006333	P	chromatin assembly or disassembly	5	56	0.0097	0.023
ChewerAug	GO:0006520	P	cellular amino acid metabolic process	15	330	0.012	0.026
ChewerAug	GO:0032318	P	regulation of Ras GTPase activity	5	59	0.012	0.027
ChewerAug	GO:0043087	P	regulation of GTPase activity	5	59	0.012	0.027
ChewerAug	GO:0007242	P	intracellular signaling cascade	7	107	0.013	0.028
ChewerAug	GO:0006281	P	DNA repair	9	163	0.015	0.031
ChewerAug	GO:0006629	P	lipid metabolic process	27	731	0.015	0.031
ChewerAug	GO:0006974	P	response to DNA damage stimulus	9	164	0.015	0.031
ChewerAug	GO:0033554	P	cellular response to stress	9	164	0.015	0.031
ChewerAug	GO:0051716	P	cellular response to stimulus	9	164	0.015	0.031
ChewerAug	GO:0051336	P	regulation of hydrolase activity	5	63	0.016	0.032
ChewerAug	GO:0005975	P	carbohydrate metabolic process	38	1136	0.02	0.039
ChewerAug	GO:0019318	P	hexose metabolic process	9	171	0.019	0.039
ChewerAug	GO:0006754	P	ATP biosynthetic process	9	173	0.021	0.04
ChewerAug	GO:0046034	P	ATP metabolic process	9	173	0.021	0.04
ChewerAug	GO:0044275	P	cellular carbohydrate catabolic process	7	119	0.022	0.041
ChewerAug	GO:0046164	P	alcohol catabolic process	7	119	0.022	0.041
ChewerAug	GO:0034641	P	cellular nitrogen compound metabolic process	20	521	0.022	0.042
ChewerAug	GO:0005996	P	monosaccharide metabolic process	9	178	0.024	0.045
ChewerAug	GO:0006006	P	glucose metabolic process	8	151	0.026	0.045
ChewerAug	GO:0009142	P	nucleoside triphosphate biosynthetic process	9	181	0.027	0.045
ChewerAug	GO:0009144	P	purine nucleoside triphosphate metabolic process	9	181	0.027	0.045
ChewerAug	GO:0009145	P	purine nucleoside triphosphate biosynthetic process	9	181	0.027	0.045
ChewerAug	GO:0009199	P	ribonucleoside triphosphate metabolic process	9	181	0.027	0.045
ChewerAug	GO:0009201	P	ribonucleoside triphosphate biosynthetic process	9	181	0.027	0.045
ChewerAug	GO:0009205	P	purine ribonucleoside triphosphate metabolic process	9	181	0.027	0.045
ChewerAug	GO:0009206	P	purine ribonucleoside triphosphate biosynthetic process	9	181	0.027	0.045

Trait	GO term	Ontology	Description	N in QTL	N in genome	p	FDR
ChewerAug	GO:0016070	P	RNA metabolic process	46	1447	0.025	0.045
ChewerAug	GO:0032774	P	RNA biosynthetic process	36	1083	0.024	0.045
ChewerAug	GO:0006325	P	chromatin organization	6	99	0.028	0.048
ChewerAug	GO:0006355	P	regulation of transcription, DNA-dependent	34	1027	0.029	0.048
ChewerAug	GO:0009165	P	nucleotide biosynthetic process	11	244	0.029	0.048
ChewerAug	GO:0051252	P	regulation of RNA metabolic process	34	1027	0.029	0.048
ChewerAug	GO:0009141	P	nucleoside triphosphate metabolic process	9	185	0.03	0.049
LeafSuckAug	GO:0008152	P	metabolic process	596	12037	3.30E-30	6.80E-28
LeafSuckAug	GO:0044238	P	primary metabolic process	467	9502	2.00E-20	2.00E-18
LeafSuckAug	GO:0043170	P	macromolecule metabolic process	368	7539	1.10E-14	7.80E-13
LeafSuckAug	GO:0044237	P	cellular metabolic process	401	8506	6.30E-14	3.20E-12
LeafSuckAug	GO:0044260	P	cellular macromolecule metabolic process	331	6824	1.30E-12	5.20E-11
LeafSuckAug	GO:0009987	P	cellular process	468	10572	3.00E-12	1.00E-10
LeafSuckAug	GO:0006807	P	nitrogen compound metabolic process	203	3784	4.90E-11	1.40E-09
LeafSuckAug	GO:0006139	P	nucleobase, nucleoside, nucleotide and nucleic acid metabolic process	176	3290	1.50E-09	4.00E-08
LeafSuckAug	GO:0009058	P	biosynthetic process	207	4271	8.70E-08	2.00E-06
LeafSuckAug	GO:0010467	P	gene expression	144	2807	6.20E-07	1.30E-05
LeafSuckAug	GO:0034645	P	cellular macromolecule biosynthetic process	154	3130	2.40E-06	4.50E-05
LeafSuckAug	GO:0009059	P	macromolecule biosynthetic process	154	3136	2.70E-06	4.60E-05
LeafSuckAug	GO:0044249	P	cellular biosynthetic process	187	4007	4.40E-06	6.90E-05
LeafSuckAug	GO:0051179	P	localization	103	1942	7.70E-06	0.00011
LeafSuckAug	GO:0051234	P	establishment of localization	100	1911	1.80E-05	0.00022
LeafSuckAug	GO:0006350	P	transcription	98	1864	1.80E-05	0.00022
LeafSuckAug	GO:0006810	P	transport	100	1911	1.80E-05	0.00022
LeafSuckAug	GO:0019538	P	protein metabolic process	199	4499	4.90E-05	0.00056
LeafSuckAug	GO:0016070	P	RNA metabolic process	78	1447	6.10E-05	0.00066
LeafSuckAug	GO:0030001	P	metal ion transport	24	285	8.70E-05	0.00085
LeafSuckAug	GO:0005975	P	carbohydrate metabolic process	64	1136	8.70E-05	0.00085
LeafSuckAug	GO:0009889	P	regulation of biosynthetic process	88	1720	0.00012	0.00097
LeafSuckAug	GO:0031326	P	regulation of cellular biosynthetic process	88	1720	0.00012	0.00097
LeafSuckAug	GO:0010556	P	regulation of macromolecule biosynthetic process	88	1720	0.00012	0.00097
LeafSuckAug	GO:0019219	P	regulation of nucleobase, nucleoside, nucleotide and nucleic acid metabolic process	88	1717	0.00011	0.00097
LeafSuckAug	GO:0045449	P	regulation of transcription	87	1713	0.00016	0.0013
LeafSuckAug	GO:0031323	P	regulation of cellular metabolic process	88	1743	0.00018	0.0014
LeafSuckAug	GO:0080090	P	regulation of primary metabolic process	91	1821	0.0002	0.0014
LeafSuckAug	GO:0051171	P	regulation of nitrogen compound metabolic process	88	1755	0.00022	0.0016
LeafSuckAug	GO:0010468	P	regulation of gene expression	87	1734	0.00024	0.0016
LeafSuckAug	GO:0060255	P	regulation of macromolecule metabolic process	91	1837	0.00026	0.0017
LeafSuckAug	GO:0006259	P	DNA metabolic process	41	688	0.00056	0.0036
LeafSuckAug	GO:0019222	P	regulation of metabolic process	91	1898	0.0007	0.0043
LeafSuckAug	GO:0034641	P	cellular nitrogen compound metabolic process	33	521	0.00072	0.0044
LeafSuckAug	GO:0006811	P	ion transport	37	624	1.10E-03	6.30E-03
LeafSuckAug	GO:0050794	P	regulation of cellular process	107	2336	0.0011	0.0063
LeafSuckAug	GO:0009056	P	catabolic process	56	1073	0.0013	0.0073
LeafSuckAug	GO:0065007	P	biological regulation	116	2601	0.0016	0.0087
LeafSuckAug	GO:0006508	P	proteolysis	44	799	0.0016	0.0087
LeafSuckAug	GO:0008104	P	protein localization	23	337	0.0018	0.0093
LeafSuckAug	GO:0050789	P	regulation of biological process	111	2490	0.0021	0.01
LeafSuckAug	GO:0006812	P	cation transport	31	513	0.0021	0.01
LeafSuckAug	GO:0009057	P	macromolecule catabolic process	51	976	0.0021	0.01
LeafSuckAug	GO:0044267	P	cellular protein metabolic process	162	3849	0.0022	0.01
LeafSuckAug	GO:0006396	P	RNA processing	18	241	0.0022	0.01

Trait	GO term	Ontology	Description	N in QTL	N in genome	p	FDR
LeafSuckAug	GO:0042545	P	cell wall modification	10	97	0.0026	0.012
LeafSuckAug	GO:0033036	P	macromolecule localization	23	353	0.0031	0.014
LeafSuckAug	GO:0006351	P	transcription, DNA-dependent	54	1079	0.0036	0.016
LeafSuckAug	GO:0032774	P	RNA biosynthetic process	54	1083	0.0039	0.016
LeafSuckAug	GO:0000160	P	two-component signal transduction system (phosphorelay)	11	123	0.0045	0.018
LeafSuckAug	GO:0045184	P	establishment of protein localization	20	303	0.0049	0.019
LeafSuckAug	GO:0015031	P	protein transport	20	303	0.0049	0.019
LeafSuckAug	GO:0006612	P	protein targeting to membrane	5	30	0.0052	0.02
LeafSuckAug	GO:0006694	P	steroid biosynthetic process	5	31	0.0059	0.022
LeafSuckAug	GO:0018130	P	heterocycle biosynthetic process	11	130	0.0065	0.024
LeafSuckAug	GO:0016071	P	mRNA metabolic process	5	33	0.0075	0.027
LeafSuckAug	GO:0006397	P	mRNA processing	5	33	0.0075	0.027
LeafSuckAug	GO:0006313	P	transposition, DNA-mediated	8	80	0.008	0.028
LeafSuckAug	GO:0032196	P	transposition	8	80	0.008	0.028
LeafSuckAug	GO:0044271	P	cellular nitrogen compound biosynthetic process	19	298	0.0084	0.029
LeafSuckAug	GO:0006520	P	cellular amino acid metabolic process	20	330	0.011	0.038
LeafSuckAug	GO:0006355	P	regulation of transcription, DNA-dependent	49	1027	0.012	0.039
LeafSuckAug	GO:0051641	P	cellular localization	21	354	0.012	0.039
LeafSuckAug	GO:0051252	P	regulation of RNA metabolic process	49	1027	0.012	0.039
LeafSuckAug	GO:0051716	P	cellular response to stimulus	12	164	0.013	0.04
LeafSuckAug	GO:0033554	P	cellular response to stress	12	164	0.013	0.04
LeafSuckAug	GO:0019438	P	aromatic compound biosynthetic process	7	71	0.014	0.04
LeafSuckAug	GO:0006461	P	protein complex assembly	11	145	0.013	0.04
LeafSuckAug	GO:0070271	P	protein complex biogenesis	11	145	0.013	0.04
LeafSuckAug	GO:0006996	P	organelle organization	14	205	0.013	0.04
LeafSuckAug	GO:0044085	P	cellular component biogenesis	16	253	0.016	0.045
LeafSuckAug	GO:0006310	P	DNA recombination	8	92	0.016	0.047
LeafSuckAug	GO:0044106	P	cellular amine metabolic process	20	345	0.017	0.048
LeafSuckAug	GO:0022607	P	cellular component assembly	13	193	0.018	0.05
LeafSuckAug	GO:0043436	P	oxoacid metabolic process	27	511	0.018	0.05
LeafSuckAug	GO:0019752	P	carboxylic acid metabolic process	27	511	0.018	0.05
LeafSuckAug	GO:0006352	P	transcription initiation	6	59	0.019	0.05
LeafSuckAug	GO:0006082	P	organic acid metabolic process	27	512	0.019	0.05
LeafSuckAug	GO:0003824	F	catalytic activity	609	12409	3.80E-30	5.80E-28
LeafSuckAug	GO:0005488	F	binding	598	12383	2.80E-27	2.10E-25
LeafSuckAug	GO:0016787	F	hydrolase activity	210	3729	2.70E-13	1.40E-11
LeafSuckAug	GO:0003676	F	nucleic acid binding	183	3583	1.90E-08	7.10E-07
LeafSuckAug	GO:0005515	F	protein binding	128	2351	1.50E-07	3.50E-06
LeafSuckAug	GO:0046872	F	metal ion binding	137	2557	1.20E-07	3.50E-06
LeafSuckAug	GO:0043169	F	cation binding	137	2576	1.80E-07	3.50E-06
LeafSuckAug	GO:0043167	F	ion binding	137	2576	1.80E-07	3.50E-06
LeafSuckAug	GO:0046914	F	transition metal ion binding	114	2131	1.70E-06	2.80E-05
LeafSuckAug	GO:0003677	F	DNA binding	104	2000	1.50E-05	0.00023
LeafSuckAug	GO:0004185	F	serine-type carboxypeptidase activity	12	73	2.00E-05	0.00025
LeafSuckAug	GO:0070008	F	serine-type exopeptidase activity	12	73	2.00E-05	0.00025
LeafSuckAug	GO:0016740	F	transferase activity	210	4741	2.70E-05	0.00031
LeafSuckAug	GO:0016491	F	oxidoreductase activity	120	2435	3.30E-05	0.00036
LeafSuckAug	GO:0004180	F	carboxypeptidase activity	12	80	4.40E-05	0.00045
LeafSuckAug	GO:0046873	F	metal ion transmembrane transporter activity	15	126	5.90E-05	0.00057
LeafSuckAug	GO:0016817	F	hydrolase activity, acting on acid anhydrides	64	1136	8.70E-05	0.00079
LeafSuckAug	GO:0016818	F	hydrolase activity, acting on acid anhydrides, in phosphorus-containing anhydrides	61	1077	0.00011	0.00089
LeafSuckAug	GO:0008238	F	exopeptidase activity	12	89	0.00011	0.00089

Trait	GO term	Ontology	Description	N in QTL	N in genome	p	FDR
LeafSuckAug	GO:0017171	F	serine hydrolase activity	23	282	0.00019	0.0013
LeafSuckAug	GO:0008236	F	serine-type peptidase activity	23	282	0.00019	0.0013
LeafSuckAug	GO:0016462	F	pyrophosphatase activity	59	1052	0.00018	0.0013
LeafSuckAug	GO:0016887	F	ATPase activity	40	633	0.00022	0.0015
LeafSuckAug	GO:0030528	F	transcription regulator activity	72	1374	0.00026	0.0016
LeafSuckAug	GO:0008270	F	zinc ion binding	63	1165	0.00028	0.0017
LeafSuckAug	GO:0017111	F	nucleoside-triphosphatase activity	57	1030	0.00032	0.0019
LeafSuckAug	GO:0004553	F	hydrolase activity, hydrolyzing O-glycosyl compounds	37	593	0.00046	0.0026
LeafSuckAug	GO:0070011	F	peptidase activity, acting on L-amino acid peptides	40	676	0.00074	0.0041
LeafSuckAug	GO:0017076	F	purine nucleotide binding	205	4945	0.0011	0.0053
LeafSuckAug	GO:0032555	F	purine ribonucleotide binding	196	4698	0.0011	0.0053
LeafSuckAug	GO:0032553	F	ribonucleotide binding	196	4698	0.0011	0.0053
LeafSuckAug	GO:0016798	F	hydrolase activity, acting on glycosyl bonds	37	623	0.0011	0.0053
LeafSuckAug	GO:0016772	F	transferase activity, transferring phosphorus-containing groups	136	3097	0.0012	0.0054
LeafSuckAug	GO:0016758	F	transferase activity, transferring hexosyl groups	34	562	0.0013	0.0057
LeafSuckAug	GO:0008233	F	peptidase activity	40	699	0.0013	0.0058
LeafSuckAug	GO:0000166	F	nucleotide binding	209	5094	0.0016	0.0068
LeafSuckAug	GO:0016788	F	hydrolase activity, acting on ester bonds	45	830	0.0019	0.0079
LeafSuckAug	GO:0032559	F	adenyl ribonucleotide binding	183	4429	0.0024	0.0095
LeafSuckAug	GO:0005524	F	ATP binding	183	4427	0.0024	0.0095
LeafSuckAug	GO:0001883	F	purine nucleoside binding	191	4664	0.0028	0.01
LeafSuckAug	GO:0001882	F	nucleoside binding	191	4666	0.0029	0.01
LeafSuckAug	GO:0030554	F	adenyl nucleotide binding	191	4664	0.0028	0.01
LeafSuckAug	GO:0005507	F	copper ion binding	14	171	0.0031	0.011
LeafSuckAug	GO:0030599	F	pectinesterase activity	12	136	0.0034	0.012
LeafSuckAug	GO:0004091	F	carboxylesterase activity	16	212	0.0034	0.012
LeafSuckAug	GO:0009055	F	electron carrier activity	46	893	0.0043	0.014
LeafSuckAug	GO:0004857	F	enzyme inhibitor activity	13	164	0.0055	0.018
LeafSuckAug	GO:0016779	F	nucleotidyltransferase activity	29	507	0.0058	0.018
LeafSuckAug	GO:0003700	F	transcription factor activity	45	891	0.0065	0.02
LeafSuckAug	GO:0022892	F	substrate-specific transporter activity	36	683	0.0077	0.023
LeafSuckAug	GO:0005215	F	transporter activity	64	1378	0.0079	0.023
LeafSuckAug	GO:0004803	F	transposase activity	8	80	0.008	0.023
LeafSuckAug	GO:0016884	F	carbon-nitrogen ligase activity, with glutamine as amido-N-donor	5	34	0.0083	0.024
LeafSuckAug	GO:0003887	F	DNA-directed DNA polymerase activity	5	35	0.0093	0.026
LeafSuckAug	GO:0008237	F	metallopeptidase activity	10	121	0.011	0.03
LeafSuckAug	GO:0016757	F	transferase activity, transferring glycosyl groups	35	678	0.011	0.03
LeafSuckAug	GO:0016627	F	oxidoreductase activity, acting on the CH-CH group of donors	7	68	0.011	0.03
LeafSuckAug	GO:0005543	F	phospholipid binding	6	53	0.012	0.032
LeafSuckAug	GO:0000156	F	two-component response regulator activity	9	105	0.012	0.032
LeafSuckAug	GO:0030234	F	enzyme regulator activity	19	311	0.012	0.032
LeafSuckAug	GO:0051082	F	unfolded protein binding	7	72	0.014	0.036
LeafSuckAug	GO:0016616	F	oxidoreductase activity, acting on the CH-OH group of donors, NAD or NADP as accepto	12	167	0.015	0.036
LeafSuckAug	GO:0046906	F	tetrapyrrole binding	35	699	0.017	0.041
LeafSuckAug	GO:0016614	F	oxidoreductase activity, acting on CH-OH group of donors	13	192	0.017	0.041
LeafSuckAug	GO:0016773	F	phosphotransferase activity, alcohol group as acceptor	103	2473	0.018	0.042
LeafSuckAug	GO:0015079	F	potassium ion transmembrane transporter activity	5	43	0.02	0.045
LeafSuckAug	GO:0034061	F	DNA polymerase activity	19	329	0.02	0.047
LeafSuckAug	GO:0020037	F	heme binding	34	689	0.022	0.049
LeafSuckAug	GO:0008324	F	cation transmembrane transporter activity	19	332	0.022	0.049
LeafSuckAug	GO:0044464	C	cell part	360	7109	1.30E-16	3.00E-15
LeafSuckAug	GO:0005623	C	cell	360	7109	1.30E-16	3.00E-15

Trait	GO term	Ontology	Description	N in QTL	N in genome	p	FDR
LeafSuckAug	GO:0005622	C	intracellular	215	4246	1.50E-09	2.20E-08
LeafSuckAug	GO:0043231	C	intracellular membrane-bounded organelle	114	2042	2.30E-07	1.70E-06
LeafSuckAug	GO:0043229	C	intracellular organelle	147	2821	2.00E-07	1.70E-06
LeafSuckAug	GO:0043226	C	organelle	147	2821	2.00E-07	1.70E-06
LeafSuckAug	GO:0043227	C	membrane-bounded organelle	114	2055	3.10E-07	2.00E-06
LeafSuckAug	GO:0044424	C	intracellular part	174	3536	4.70E-07	2.60E-06
LeafSuckAug	GO:0005634	C	nucleus	89	1626	1.10E-05	5.30E-05
LeafSuckAug	GO:0016020	C	membrane	151	3219	3.30E-05	0.00015
LeafSuckAug	GO:0044429	C	mitochondrial part	14	110	5.40E-05	0.00022
LeafSuckAug	GO:0005739	C	mitochondrion	14	118	0.00011	0.00039
LeafSuckAug	GO:0044422	C	organelle part	43	680	0.00013	0.0004
LeafSuckAug	GO:0044446	C	intracellular organelle part	43	680	0.00013	0.0004
LeafSuckAug	GO:0005681	C	spliceosomal complex	5	12	0.00016	0.00047
LeafSuckAug	GO:0044428	C	nuclear part	14	132	0.00031	0.00085
LeafSuckAug	GO:0005740	C	mitochondrial envelope	11	96	0.00074	0.0017
LeafSuckAug	GO:0031967	C	organelle envelope	14	145	0.00073	0.0017
LeafSuckAug	GO:0032991	C	macromolecular complex	75	1499	0.00068	0.0017
LeafSuckAug	GO:0031974	C	membrane-enclosed lumen	9	70	0.0011	0.0023
LeafSuckAug	GO:0044444	C	cytoplasmic part	55	1085	0.0026	0.0055
LeafSuckAug	GO:0070013	C	intracellular organelle lumen	7	61	0.0066	0.012
LeafSuckAug	GO:0016021	C	integral to membrane	53	1082	0.0059	0.012
LeafSuckAug	GO:0043233	C	organelle lumen	7	61	0.0066	0.012
LeafSuckAug	GO:0015629	C	actin cytoskeleton	5	32	0.0067	0.012
LeafSuckAug	GO:0005737	C	cytoplasm	65	1393	0.0068	0.012
LeafSuckAug	GO:0030529	C	ribonucleoprotein complex	28	512	0.011	0.018
LeafSuckAug	GO:0031975	C	envelope	14	204	0.013	0.02
LeafSuckAug	GO:0043234	C	protein complex	46	965	0.015	0.023
LeafSuckAug	GO:0005694	C	chromosome	10	139	0.024	0.036
LeafSuckAug	GO:0005618	C	cell wall	11	164	0.029	0.041
LeafSuckAug	GO:0043232	C	intracellular non-membrane-bounded organelle	37	797	0.037	0.049
LeafSuckAug	GO:0043228	C	non-membrane-bounded organelle	37	797	0.037	0.049
MinerAug	GO:0008152	P	metabolic process	680	12037	1.10E-29	2.30E-27
MinerAug	GO:0009987	P	cellular process	594	10572	1.40E-23	1.50E-21
MinerAug	GO:0044238	P	primary metabolic process	537	9502	4.90E-21	3.50E-19
MinerAug	GO:0043170	P	macromolecule metabolic process	432	7539	6.20E-17	3.30E-15
MinerAug	GO:0044237	P	cellular metabolic process	474	8506	9.40E-17	4.00E-15
MinerAug	GO:0044260	P	cellular macromolecule metabolic process	391	6824	4.80E-15	1.70E-13
MinerAug	GO:0019538	P	protein metabolic process	262	4499	1.80E-10	5.40E-09
MinerAug	GO:0044267	P	cellular protein metabolic process	226	3849	2.30E-09	5.40E-08
MinerAug	GO:0050794	P	regulation of cellular process	153	2336	2.10E-09	5.40E-08
MinerAug	GO:0009058	P	biosynthetic process	242	4271	1.10E-08	2.30E-07
MinerAug	GO:0050789	P	regulation of biological process	157	2490	1.40E-08	2.80E-07
MinerAug	GO:0065007	P	biological regulation	161	2601	2.90E-08	5.20E-07
MinerAug	GO:0006807	P	nitrogen compound metabolic process	214	3784	1.10E-07	1.70E-06
MinerAug	GO:0044249	P	cellular biosynthetic process	224	4007	1.20E-07	1.70E-06
MinerAug	GO:0010467	P	gene expression	168	2807	1.20E-07	1.70E-06
MinerAug	GO:0031323	P	regulation of cellular metabolic process	115	1743	1.80E-07	2.40E-06
MinerAug	GO:0009889	P	regulation of biosynthetic process	113	1720	2.80E-07	2.80E-06
MinerAug	GO:0031326	P	regulation of cellular biosynthetic process	113	1720	2.80E-07	2.80E-06
MinerAug	GO:0045449	P	regulation of transcription	113	1713	2.30E-07	2.80E-06
MinerAug	GO:0010556	P	regulation of macromolecule biosynthetic process	113	1720	2.80E-07	2.80E-06
MinerAug	GO:0019219	P	regulation of nucleobase, nucleoside, nucleotide and nucleic acid metabolic process	113	1717	2.60E-07	2.80E-06

Trait	GO term	Ontology	Description	N in QTL	N in genome	p	FDR
MinerAug	GO:0006350	P	transcription	120	1864	3.10E-07	3.10E-06
MinerAug	GO:0006950	P	response to stress	56	660	3.30E-07	3.10E-06
MinerAug	GO:0010468	P	regulation of gene expression	113	1734	4.10E-07	3.60E-06
MinerAug	GO:0080090	P	regulation of primary metabolic process	117	1821	4.90E-07	4.00E-06
MinerAug	GO:0034645	P	cellular macromolecule biosynthetic process	180	3130	4.90E-07	4.00E-06
MinerAug	GO:0050896	P	response to stimulus	68	883	5.30E-07	4.20E-06
MinerAug	GO:0009059	P	macromolecule biosynthetic process	180	3136	5.50E-07	4.20E-06
MinerAug	GO:0051171	P	regulation of nitrogen compound metabolic process	113	1755	7.00E-07	5.20E-06
MinerAug	GO:0060255	P	regulation of macromolecule metabolic process	117	1837	7.30E-07	5.20E-06
MinerAug	GO:0006139	P	nucleobase, nucleoside, nucleotide and nucleic acid metabolic process	186	3290	8.90E-07	6.10E-06
MinerAug	GO:0002376	P	immune system process	23	167	1.00E-06	6.60E-06
MinerAug	GO:0006955	P	immune response	23	167	1.00E-06	6.60E-06
MinerAug	GO:0019222	P	regulation of metabolic process	119	1898	1.20E-06	7.70E-06
MinerAug	GO:0006464	P	protein modification process	162	2799	1.40E-06	8.50E-06
MinerAug	GO:0043412	P	macromolecule modification	165	2866	1.50E-06	8.80E-06
MinerAug	GO:0045087	P	innate immune response	22	162	2.10E-06	1.20E-05
MinerAug	GO:0006952	P	defense response	25	208	3.30E-06	1.80E-05
MinerAug	GO:0043687	P	post-translational protein modification	147	2529	3.60E-06	2.00E-05
MinerAug	GO:0016310	P	phosphorylation	139	2409	9.70E-06	5.20E-05
MinerAug	GO:0006468	P	protein amino acid phosphorylation	132	2267	1.10E-05	5.70E-05
MinerAug	GO:0006796	P	phosphate metabolic process	141	2462	1.20E-05	5.90E-05
MinerAug	GO:0006793	P	phosphorus metabolic process	141	2462	1.20E-05	5.90E-05
MinerAug	GO:0016265	P	death	44	569	4.80E-05	0.00023
MinerAug	GO:0008219	P	cell death	44	569	4.80E-05	0.00023
MinerAug	GO:0012501	P	programmed cell death	42	540	6.20E-05	0.00028
MinerAug	GO:0006915	P	apoptosis	42	540	6.20E-05	0.00028
MinerAug	GO:0051179	P	localization	111	1942	0.00011	0.0005
MinerAug	GO:0006810	P	transport	109	1911	0.00014	0.0006
MinerAug	GO:0051234	P	establishment of localization	109	1911	0.00014	0.0006
MinerAug	GO:0016070	P	RNA metabolic process	84	1447	0.00049	0.0021
MinerAug	GO:0007018	P	microtubule-based movement	13	105	0.00055	0.0023
MinerAug	GO:0006508	P	proteolysis	52	799	0.00058	0.0023
MinerAug	GO:0032774	P	RNA biosynthetic process	66	1083	0.0006	0.0024
MinerAug	GO:0009056	P	catabolic process	65	1073	0.00076	0.003
MinerAug	GO:0006351	P	transcription, DNA-dependent	65	1079	0.00087	0.0033
MinerAug	GO:0046417	P	chorismate metabolic process	6	25	0.00096	0.0035
MinerAug	GO:0009073	P	aromatic amino acid family biosynthetic process	6	25	0.00096	0.0035
MinerAug	GO:0006355	P	regulation of transcription, DNA-dependent	61	1027	0.0017	0.006
MinerAug	GO:0051252	P	regulation of RNA metabolic process	61	1027	0.0017	0.006
MinerAug	GO:0044106	P	cellular amine metabolic process	26	345	0.0022	0.0076
MinerAug	GO:0006519	P	cellular amino acid and derivative metabolic process	27	365	0.0023	0.008
MinerAug	GO:0006520	P	cellular amino acid metabolic process	25	330	0.0024	0.0083
MinerAug	GO:0034641	P	cellular nitrogen compound metabolic process	35	521	0.0026	0.0088
MinerAug	GO:0043648	P	dicarboxylic acid metabolic process	6	33	0.0033	0.011
MinerAug	GO:0007017	P	microtubule-based process	13	131	0.0034	0.011
MinerAug	GO:0042180	P	cellular ketone metabolic process	34	516	0.004	0.013
MinerAug	GO:0009072	P	aromatic amino acid family metabolic process	6	36	0.0048	0.015
MinerAug	GO:0009057	P	macromolecule catabolic process	56	976	0.005	0.016
MinerAug	GO:0043436	P	oxoacid metabolic process	33	511	0.006	0.017
MinerAug	GO:0006511	P	ubiquitin-dependent protein catabolic process	16	191	0.0058	0.017
MinerAug	GO:0051603	P	proteolysis involved in cellular protein catabolic process	16	191	0.0058	0.017
MinerAug	GO:0019941	P	modification-dependent protein catabolic process	16	191	0.0058	0.017

Trait	GO term	Ontology	Description	N in QTL	N in genome	p	FDR
MinerAug	GO:0044257	P	cellular protein catabolic process	16	191	0.0058	0.017
MinerAug	GO:0019752	P	carboxylic acid metabolic process	33	511	0.006	0.017
MinerAug	GO:0043632	P	modification-dependent macromolecule catabolic process	16	191	0.0058	0.017
MinerAug	GO:0006082	P	organic acid metabolic process	33	512	0.0061	0.017
MinerAug	GO:0044271	P	cellular nitrogen compound biosynthetic process	21	298	0.011	0.03
MinerAug	GO:0016998	P	cell wall macromolecule catabolic process	7	59	0.012	0.034
MinerAug	GO:0009308	P	amine metabolic process	26	404	0.014	0.037
MinerAug	GO:0006979	P	response to oxidative stress	13	161	0.016	0.042
MinerAug	GO:0009309	P	amine biosynthetic process	13	163	0.017	0.045
MinerAug	GO:0044265	P	cellular macromolecule catabolic process	21	314	0.018	0.046
MinerAug	GO:0044036	P	cell wall macromolecule metabolic process	7	65	0.019	0.049
MinerAug	GO:0008652	P	cellular amino acid biosynthetic process	12	148	0.019	0.049
MinerAug	GO:0005488	F	binding	727	12383	4.60E-39	7.50E-37
MinerAug	GO:0003824	F	catalytic activity	702	12409	1.80E-31	1.50E-29
MinerAug	GO:0000166	F	nucleotide binding	306	5094	7.30E-14	4.00E-12
MinerAug	GO:0032559	F	adenyl ribonucleotide binding	272	4429	2.80E-13	9.10E-12
MinerAug	GO:0005524	F	ATP binding	272	4427	2.70E-13	9.10E-12
MinerAug	GO:0032555	F	purine ribonucleotide binding	284	4698	4.10E-13	9.50E-12
MinerAug	GO:0032553	F	ribonucleotide binding	284	4698	4.10E-13	9.50E-12
MinerAug	GO:0017076	F	purine nucleotide binding	294	4945	8.90E-13	1.30E-11
MinerAug	GO:0001883	F	purine nucleoside binding	281	4664	8.20E-13	1.30E-11
MinerAug	GO:0001882	F	nucleoside binding	281	4666	8.60E-13	1.30E-11
MinerAug	GO:0030554	F	adenyl nucleotide binding	281	4664	8.20E-13	1.30E-11
MinerAug	GO:0003676	F	nucleic acid binding	227	3583	2.70E-12	3.60E-11
MinerAug	GO:0016740	F	transferase activity	277	4741	3.10E-11	3.90E-10
MinerAug	GO:0003677	F	DNA binding	133	2000	1.20E-08	1.30E-07
MinerAug	GO:0016787	F	hydrolase activity	210	3729	2.00E-07	2.00E-06
MinerAug	GO:0004872	F	receptor activity	32	270	2.00E-07	2.00E-06
MinerAug	GO:0004888	F	transmembrane receptor activity	30	247	2.90E-07	2.80E-06
MinerAug	GO:0043565	F	sequence-specific DNA binding	51	606	1.40E-06	1.30E-05
MinerAug	GO:0005515	F	protein binding	138	2351	4.60E-06	4.00E-05
MinerAug	GO:0016772	F	transferase activity, transferring phosphorus-containing groups	172	3097	6.60E-06	5.30E-05
MinerAug	GO:0016301	F	kinase activity	146	2541	7.00E-06	5.40E-05
MinerAug	GO:0016773	F	phosphotransferase activity, alcohol group as acceptor	142	2473	9.70E-06	6.60E-05
MinerAug	GO:0003700	F	transcription factor activity	64	891	9.30E-06	6.60E-05
MinerAug	GO:0060089	F	molecular transducer activity	41	479	1.00E-05	6.60E-05
MinerAug	GO:0004871	F	signal transducer activity	41	479	1.00E-05	6.60E-05
MinerAug	GO:0004672	F	protein kinase activity	133	2301	1.40E-05	8.90E-05
MinerAug	GO:0030528	F	transcription regulator activity	85	1374	6.50E-05	0.00039
MinerAug	GO:0004713	F	protein tyrosine kinase activity	80	1300	0.00012	0.00073
MinerAug	GO:0043169	F	cation binding	138	2576	0.00026	0.0014
MinerAug	GO:0043167	F	ion binding	138	2576	0.00026	0.0014
MinerAug	GO:0046872	F	metal ion binding	137	2557	0.00028	0.0014
MinerAug	GO:0016765	F	transferase activity, transferring alkyl or aryl (other than methyl) groups	10	67	0.00065	0.0033
MinerAug	GO:0017111	F	nucleoside-triphosphatase activity	63	1030	0.00073	0.0036
MinerAug	GO:0016462	F	pyrophosphatase activity	64	1052	0.00076	0.0036
MinerAug	GO:0016818	F	hydrolase activity, acting on acid anhydrides, in phosphorus-containing anhydrides	64	1077	0.0013	0.006
MinerAug	GO:0016887	F	ATPase activity	42	633	0.0013	0.006
MinerAug	GO:0003777	F	microtubule motor activity	13	119	0.0016	0.007
MinerAug	GO:0016491	F	oxidoreductase activity	126	2435	0.0016	0.0071
MinerAug	GO:0016817	F	hydrolase activity, acting on acid anhydrides	66	1136	0.0019	0.0076
MinerAug	GO:0046914	F	transition metal ion binding	112	2131	0.0018	0.0076

Trait	GO term	Ontology	Description	N in QTL	N in genome	p	FDR
MinerAug	GO:0016788	F	hydrolase activity, acting on ester bonds	51	830	0.002	0.008
MinerAug	GO:0070279	F	vitamin B6 binding	16	171	0.0022	0.008
MinerAug	GO:0030170	F	pyridoxal phosphate binding	16	171	0.0022	0.008
MinerAug	GO:0019904	F	protein domain specific binding	5	20	0.0022	0.008
MinerAug	GO:0008270	F	zinc ion binding	67	1165	0.0022	0.008
MinerAug	GO:0003774	F	motor activity	14	142	0.0026	0.0091
MinerAug	GO:0008066	F	glutamate receptor activity	8	65	0.0064	0.019
MinerAug	GO:0005215	F	transporter activity	74	1378	0.0065	0.019
MinerAug	GO:0015276	F	ligand-gated ion channel activity	8	65	0.0064	0.019
MinerAug	GO:0022834	F	ligand-gated channel activity	8	65	0.0064	0.019
MinerAug	GO:0019842	F	vitamin binding	16	190	0.0056	0.019
MinerAug	GO:0005230	F	extracellular ligand-gated ion channel activity	8	65	0.0064	0.019
MinerAug	GO:0005231	F	excitatory extracellular ligand-gated ion channel activity	8	65	0.0064	0.019
MinerAug	GO:0005234	F	extracellular-glutamate-gated ion channel activity	8	65	0.0064	0.019
MinerAug	GO:0004970	F	ionotropic glutamate receptor activity	8	65	0.0064	0.019
MinerAug	GO:0005216	F	ion channel activity	13	145	0.0074	0.021
MinerAug	GO:0022838	F	substrate-specific channel activity	13	145	0.0074	0.021
MinerAug	GO:0016209	F	antioxidant activity	16	197	0.0076	0.021
MinerAug	GO:0015267	F	channel activity	13	147	0.0082	0.022
MinerAug	GO:0016874	F	ligase activity	30	464	0.0083	0.022
MinerAug	GO:0022803	F	passive transmembrane transporter activity	13	147	0.0082	0.022
MinerAug	GO:0016853	F	isomerase activity	16	200	0.0086	0.023
MinerAug	GO:0016757	F	transferase activity, transferring glycosyl groups	40	678	0.011	0.028
MinerAug	GO:0004601	F	peroxidase activity	14	171	0.011	0.028
MinerAug	GO:0016684	F	oxidoreductase activity, acting on peroxide as acceptor	14	171	0.011	0.028
MinerAug	GO:0022836	F	gated channel activity	10	104	0.012	0.029
MinerAug	GO:0020037	F	heme binding	40	689	0.013	0.033
MinerAug	GO:0004197	F	cysteine-type endopeptidase activity	5	33	0.014	0.033
MinerAug	GO:0046906	F	tetrapyrrole binding	40	699	0.016	0.039
MinerAug	GO:0044464	C	cell part	426	7109	5.50E-20	1.60E-18
MinerAug	GO:0005623	C	cell	426	7109	5.50E-20	1.60E-18
MinerAug	GO:0005622	C	intracellular	269	4246	1.30E-14	2.50E-13
MinerAug	GO:0044424	C	intracellular part	228	3536	4.50E-13	6.40E-12
MinerAug	GO:0043229	C	intracellular organelle	187	2821	9.90E-12	9.40E-11
MinerAug	GO:0043226	C	organelle	187	2821	9.90E-12	9.40E-11
MinerAug	GO:0005634	C	nucleus	115	1626	5.60E-09	4.60E-08
MinerAug	GO:0043227	C	membrane-bounded organelle	136	2055	1.00E-08	7.30E-08
MinerAug	GO:0043231	C	intracellular membrane-bounded organelle	135	2042	1.20E-08	7.80E-08
MinerAug	GO:0032991	C	macromolecular complex	98	1499	2.20E-06	1.20E-05
MinerAug	GO:0043234	C	protein complex	69	965	4.90E-06	2.50E-05
MinerAug	GO:0016020	C	membrane	174	3219	2.50E-05	0.00012
MinerAug	GO:0044425	C	membrane part	95	1551	3.50E-05	0.00015
MinerAug	GO:0031224	C	intrinsic to membrane	80	1270	6.10E-05	0.00025
MinerAug	GO:0044422	C	organelle part	49	680	9.40E-05	0.00033
MinerAug	GO:0044446	C	intracellular organelle part	49	680	9.40E-05	0.00033
MinerAug	GO:0043232	C	intracellular non-membrane-bounded organelle	53	797	0.00032	0.001
MinerAug	GO:0043228	C	non-membrane-bounded organelle	53	797	0.00032	0.001
MinerAug	GO:0005737	C	cytoplasm	80	1393	0.00089	0.0027
MinerAug	GO:0016272	C	prefoldin complex	5	16	0.00096	0.0027
MinerAug	GO:0005875	C	microtubule associated complex	13	119	0.0016	0.0043
MinerAug	GO:0044428	C	nuclear part	13	132	0.0036	0.0086
MinerAug	GO:0044444	C	cytoplasmic part	62	1085	0.0036	0.0086

Trait	GO term	Ontology	Description	N in QTL	N in genome	p	FDR
MinerAug	GO:0015630	C	microtubule cytoskeleton	13	132	0.0036	0.0086
MinerAug	GO:0044430	C	cytoskeletal part	14	156	0.0055	0.013
MinerAug	GO:0030288	C	outer membrane-bounded periplasmic space	7	56	0.0097	0.021
MinerAug	GO:0042597	C	periplasmic space	7	57	0.011	0.022
MinerAug	GO:0044462	C	external encapsulating structure part	7	58	0.011	0.022
MinerAug	GO:0005856	C	cytoskeleton	15	189	0.011	0.022
MinerAug	GO:0030313	C	cell envelope	7	59	0.012	0.024
MinerAug	GO:0016021	C	integral to membrane	58	1082	0.015	0.027
MinerAug	GO:0000145	C	exocyst	6	48	0.016	0.027
MinerAug	GO:0044448	C	cell cortex part	6	48	0.016	0.027
MinerAug	GO:0005938	C	cell cortex	6	48	0.016	0.027
MinerAug	GO:0030312	C	external encapsulating structure	16	223	0.021	0.034
MinerAug	GO:0005829	C	cytosol	5	41	0.03	0.046
MinerAug	GO:0044445	C	cytosolic part	5	41	0.03	0.046
MinerJune	GO:0008152	P	metabolic process	293	12037	5.20E-17	7.20E-15
MinerJune	GO:0044238	P	primary metabolic process	233	9502	1.20E-12	8.20E-11
MinerJune	GO:0009987	P	cellular process	242	10572	4.50E-10	2.10E-08
MinerJune	GO:0044237	P	cellular metabolic process	200	8506	6.30E-09	2.20E-07
MinerJune	GO:0043170	P	macromolecule metabolic process	178	7539	5.70E-08	1.60E-06
MinerJune	GO:0044260	P	cellular macromolecule metabolic process	160	6824	6.10E-07	1.40E-05
MinerJune	GO:0005975	P	carbohydrate metabolic process	38	1136	4.10E-05	0.00082
MinerJune	GO:0006350	P	transcription	54	1864	4.90E-05	0.00085
MinerJune	GO:0009058	P	biosynthetic process	101	4271	9.90E-05	0.0015
MinerJune	GO:0006979	P	response to oxidative stress	11	161	0.00011	0.0016
MinerJune	GO:0006139	P	nucleobase, nucleoside, nucleotide and nucleic acid metabolic process	81	3290	0.00016	0.002
MinerJune	GO:0080090	P	regulation of primary metabolic process	48	1821	0.00098	0.0076
MinerJune	GO:0019222	P	regulation of metabolic process	49	1898	0.0013	0.0076
MinerJune	GO:0060255	P	regulation of macromolecule metabolic process	49	1837	0.00068	0.0076
MinerJune	GO:0019538	P	protein metabolic process	99	4499	0.0013	0.0076
MinerJune	GO:0006807	P	nitrogen compound metabolic process	87	3784	0.00078	0.0076
MinerJune	GO:0050789	P	regulation of biological process	61	2490	0.0012	0.0076
MinerJune	GO:0065007	P	biological regulation	63	2601	0.0013	0.0076
MinerJune	GO:0009889	P	regulation of biosynthetic process	45	1720	0.0016	0.0076
MinerJune	GO:0043412	P	macromolecule modification	68	2866	0.0014	0.0076
MinerJune	GO:0044249	P	cellular biosynthetic process	90	4007	0.0013	0.0076
MinerJune	GO:0030001	P	metal ion transport	13	285	0.0012	0.0076
MinerJune	GO:0031326	P	regulation of cellular biosynthetic process	45	1720	0.0016	0.0076
MinerJune	GO:0045449	P	regulation of transcription	45	1713	0.0015	0.0076
MinerJune	GO:0010467	P	gene expression	68	2807	0.00085	0.0076
MinerJune	GO:0010556	P	regulation of macromolecule biosynthetic process	45	1720	0.0016	0.0076
MinerJune	GO:0010468	P	regulation of gene expression	46	1734	0.0011	0.0076
MinerJune	GO:0019219	P	regulation of nucleobase, nucleoside, nucleotide and nucleic acid metabolic process	45	1717	0.0016	0.0076
MinerJune	GO:0006464	P	protein modification process	67	2799	0.0013	0.0076
MinerJune	GO:0009059	P	macromolecule biosynthetic process	73	3136	0.0016	0.0076
MinerJune	GO:0031323	P	regulation of cellular metabolic process	45	1743	0.0021	0.0094
MinerJune	GO:0034645	P	cellular macromolecule biosynthetic process	72	3130	0.0023	0.0099
MinerJune	GO:0051171	P	regulation of nitrogen compound metabolic process	45	1755	0.0024	0.01
MinerJune	GO:0006812	P	cation transport	18	513	0.0026	0.011
MinerJune	GO:0044262	P	cellular carbohydrate metabolic process	17	491	0.0039	0.014
MinerJune	GO:0006813	P	potassium ion transport	5	59	0.0037	0.014
MinerJune	GO:0044248	P	cellular catabolic process	14	367	0.0037	0.014
MinerJune	GO:0009056	P	catabolic process	30	1073	0.0038	0.014

Trait	GO term	Ontology	Description	N in QTL	N in genome	p	FDR
MinerJune	GO:0009057	P	macromolecule catabolic process	28	976	0.0037	0.014
MinerJune	GO:0042221	P	response to chemical stimulus	13	336	0.0046	0.016
MinerJune	GO:0008610	P	lipid biosynthetic process	11	259	0.0046	0.016
MinerJune	GO:0050794	P	regulation of cellular process	55	2336	0.0047	0.016
MinerJune	GO:0044267	P	cellular protein metabolic process	83	3849	0.0057	0.019
MinerJune	GO:0006468	P	protein amino acid phosphorylation	53	2267	0.0063	0.02
MinerJune	GO:0044265	P	cellular macromolecule catabolic process	12	314	0.0069	0.021
MinerJune	GO:0006355	P	regulation of transcription, DNA-dependent	28	1027	0.0071	0.021
MinerJune	GO:0051252	P	regulation of RNA metabolic process	28	1027	0.0071	0.021
MinerJune	GO:0006351	P	transcription, DNA-dependent	29	1079	0.0074	0.022
MinerJune	GO:0032774	P	RNA biosynthetic process	29	1083	0.0078	0.022
MinerJune	GO:0006811	P	ion transport	19	624	0.0086	0.024
MinerJune	GO:0051179	P	localization	46	1942	0.0086	0.024
MinerJune	GO:0043687	P	post-translational protein modification	57	2529	0.0095	0.026
MinerJune	GO:0006810	P	transport	45	1911	0.01	0.026
MinerJune	GO:0051234	P	establishment of localization	45	1911	0.01	0.026
MinerJune	GO:0044085	P	cellular component biogenesis	10	253	0.011	0.027
MinerJune	GO:0016310	P	phosphorylation	54	2409	0.013	0.031
MinerJune	GO:0006796	P	phosphate metabolic process	55	2462	0.013	0.031
MinerJune	GO:0006793	P	phosphorus metabolic process	55	2462	0.013	0.031
MinerJune	GO:0006508	P	proteolysis	22	799	0.014	0.034
MinerJune	GO:0003824	F	catalytic activity	315	12409	3.00E-22	3.10E-20
MinerJune	GO:0005488	F	binding	306	12383	2.80E-19	1.40E-17
MinerJune	GO:0043169	F	cation binding	76	2576	6.30E-07	1.10E-05
MinerJune	GO:0000166	F	nucleotide binding	127	5094	6.70E-07	1.10E-05
MinerJune	GO:0046872	F	metal ion binding	76	2557	4.80E-07	1.10E-05
MinerJune	GO:0043167	F	ion binding	76	2576	6.30E-07	1.10E-05
MinerJune	GO:0017076	F	purine nucleotide binding	123	4945	1.20E-06	1.50E-05
MinerJune	GO:0016491	F	oxidoreductase activity	72	2435	1.20E-06	1.50E-05
MinerJune	GO:0003677	F	DNA binding	62	2000	1.70E-06	1.90E-05
MinerJune	GO:0032555	F	purine ribonucleotide binding	116	4698	3.80E-06	3.50E-05
MinerJune	GO:0032553	F	ribonucleotide binding	116	4698	3.80E-06	3.50E-05
MinerJune	GO:0030554	F	adenyl nucleotide binding	111	4664	3.10E-05	0.00022
MinerJune	GO:0016787	F	hydrolase activity	93	3729	3.00E-05	0.00022
MinerJune	GO:0001883	F	purine nucleoside binding	111	4664	3.10E-05	0.00022
MinerJune	GO:0001882	F	nucleoside binding	111	4666	3.20E-05	0.00022
MinerJune	GO:0016684	F	oxidoreductase activity, acting on peroxide as acceptor	12	171	4.40E-05	0.00025
MinerJune	GO:0004601	F	peroxidase activity	12	171	4.40E-05	0.00025
MinerJune	GO:0046914	F	transition metal ion binding	60	2131	4.00E-05	0.00025
MinerJune	GO:0030528	F	transcription regulator activity	43	1374	5.70E-05	0.00031
MinerJune	GO:0005524	F	ATP binding	105	4427	6.20E-05	0.00031
MinerJune	GO:0032559	F	adenyl ribonucleotide binding	105	4429	6.30E-05	0.00031
MinerJune	GO:0003924	F	GTPase activity	10	137	0.00014	0.00065
MinerJune	GO:0016740	F	transferase activity	109	4741	0.00015	0.00066
MinerJune	GO:0016209	F	antioxidant activity	12	197	0.00016	0.00067
MinerJune	GO:0016817	F	hydrolase activity, acting on acid anhydrides	36	1136	0.00018	0.00075
MinerJune	GO:0003676	F	nucleic acid binding	86	3583	0.00022	0.00088
MinerJune	GO:0016818	F	hydrolase activity, acting on acid anhydrides, in phosphorus-containing anhydrides	33	1077	0.00059	0.0022
MinerJune	GO:0005506	F	iron ion binding	26	780	0.00069	0.0026
MinerJune	GO:0005507	F	copper ion binding	10	171	0.00073	0.0026
MinerJune	GO:0048037	F	cofactor binding	25	774	0.0013	0.0046
MinerJune	GO:0016301	F	kinase activity	61	2541	0.0019	0.0065

Trait	GO term	Ontology	Description	N in QTL	N in genome	p	FDR
MinerJune	GO:0003700	F	transcription factor activity	27	891	0.0021	0.0067
MinerJune	GO:0017111	F	nucleoside-triphosphatase activity	30	1030	0.0022	0.0068
MinerJune	GO:0016773	F	phosphotransferase activity, alcohol group as acceptor	59	2473	0.0026	0.008
MinerJune	GO:0003899	F	DNA-directed RNA polymerase activity	6	81	0.0028	0.008
MinerJune	GO:0034062	F	RNA polymerase activity	6	81	0.0028	0.008
MinerJune	GO:0016462	F	pyrophosphatase activity	30	1052	0.0029	0.0081
MinerJune	GO:0050662	F	coenzyme binding	19	572	0.0036	0.0098
MinerJune	GO:0019001	F	guanyl nucleotide binding	12	291	0.0039	0.01
MinerJune	GO:0008324	F	cation transmembrane transporter activity	13	332	0.0042	0.011
MinerJune	GO:0016614	F	oxidoreductase activity, acting on CH-OH group of donors	9	192	0.0054	0.014
MinerJune	GO:0046873	F	metal ion transmembrane transporter activity	7	126	0.0058	0.014
MinerJune	GO:0020037	F	heme binding	21	689	0.0059	0.014
MinerJune	GO:0016772	F	transferase activity, transferring phosphorus-containing groups	69	3097	0.0058	0.014
MinerJune	GO:0046906	F	tetrapyrrole binding	21	699	0.0068	0.016
MinerJune	GO:0009055	F	electron carrier activity	25	893	0.0078	0.016
MinerJune	GO:0016616	F	oxidoreductase activity, acting on the CH-OH group of donors, NAD or NADP as accepto	8	167	0.0076	0.016
MinerJune	GO:0005525	F	GTP binding	11	279	0.0076	0.016
MinerJune	GO:0032561	F	guanyl ribonucleotide binding	11	279	0.0076	0.016
MinerJune	GO:0016798	F	hydrolase activity, acting on glycosyl bonds	19	623	0.0085	0.017
MinerJune	GO:0004672	F	protein kinase activity	53	2301	0.0083	0.017
MinerJune	GO:0004553	F	hydrolase activity, hydrolyzing O-glycosyl compounds	18	593	0.011	0.021
MinerJune	GO:0008289	F	lipid binding	5	81	0.013	0.024
MinerJune	GO:0016758	F	transferase activity, transferring hexosyl groups	17	562	0.013	0.025
MinerJune	GO:0004175	F	endopeptidase activity	14	429	0.013	0.025
MinerJune	GO:0016853	F	isomerase activity	8	200	0.02	0.036
MinerJune	GO:0005215	F	transporter activity	33	1378	0.021	0.038
MinerJune	GO:0043565	F	sequence-specific DNA binding	17	606	0.025	0.044
MinerJune	GO:0044464	C	cell part	174	7109	6.70E-09	9.00E-08
MinerJune	GO:0005623	C	cell	174	7109	6.70E-09	9.00E-08
MinerJune	GO:0016020	C	membrane	90	3219	4.90E-07	4.40E-06
MinerJune	GO:0016021	C	integral to membrane	36	1082	7.20E-05	0.00049
MinerJune	GO:0043229	C	intracellular organelle	69	2821	0.00061	0.0018
MinerJune	GO:0005622	C	intracellular	97	4246	0.00046	0.0018
MinerJune	GO:0043227	C	membrane-bounded organelle	54	2055	0.00051	0.0018
MinerJune	GO:0043231	C	intracellular membrane-bounded organelle	54	2042	0.00044	0.0018
MinerJune	GO:0043226	C	organelle	69	2821	0.00061	0.0018
MinerJune	GO:0031224	C	intrinsic to membrane	37	1270	0.00069	0.0019
MinerJune	GO:0005634	C	nucleus	44	1626	0.00097	0.0024
MinerJune	GO:0044425	C	membrane part	42	1551	0.0012	0.0028
MinerJune	GO:0044424	C	intracellular part	81	3536	0.0013	0.0028
MinerJune	GO:0005694	C	chromosome	7	139	0.0095	0.018
MinerJune	GO:0005783	C	endoplasmic reticulum	7	153	0.015	0.027
SkeletAug	GO:0006810	P	transport	34	1911	0.00012	0.003
SkeletAug	GO:0008152	P	metabolic process	139	12037	0.00011	0.003
SkeletAug	GO:0051234	P	establishment of localization	34	1911	0.00012	0.003
SkeletAug	GO:0042545	P	cell wall modification	7	97	4.00E-05	0.003
SkeletAug	GO:0051179	P	localization	34	1942	0.00016	0.0032
SkeletAug	GO:0009987	P	cellular process	121	10572	0.00067	0.011
SkeletAug	GO:0006644	P	phospholipid metabolic process	5	89	0.0015	0.019
SkeletAug	GO:0044238	P	primary metabolic process	109	9502	0.0014	0.019
SkeletAug	GO:0019637	P	organophosphate metabolic process	5	96	0.0021	0.023
SkeletAug	GO:0005488	F	binding	151	12383	1.20E-06	4.40E-05

Trait	GO term	Ontology	Description	N in QTL	N in genome	p	FDR
SkeletAug	GO:0016787	F	hydrolase activity	62	3729	1.20E-06	4.40E-05
SkeletAug	GO:0003824	F	catalytic activity	150	12409	2.30E-06	6.00E-05
SkeletAug	GO:0016817	F	hydrolase activity, acting on acid anhydrides	24	1136	0.00011	0.0021
SkeletAug	GO:0004091	F	carboxylesterase activity	9	212	0.00017	0.0025
SkeletAug	GO:0030599	F	pectinesterase activity	7	136	0.00029	0.0035
SkeletAug	GO:0005515	F	protein binding	38	2351	0.00032	0.0035
SkeletAug	GO:0016818	F	hydrolase activity, acting on acid anhydrides, in phosphorus-containing anhydrides	21	1077	0.00083	0.0079
SkeletAug	GO:0017111	F	nucleoside-triphosphatase activity	20	1030	0.0012	0.0097
SkeletAug	GO:0016462	F	pyrophosphatase activity	20	1052	0.0015	0.011
SkeletAug	GO:0032555	F	purine ribonucleotide binding	60	4698	0.0025	0.016
SkeletAug	GO:0032553	F	ribonucleotide binding	60	4698	0.0025	0.016
SkeletAug	GO:0000166	F	nucleotide binding	63	5094	0.0041	0.021
SkeletAug	GO:0016788	F	hydrolase activity, acting on ester bonds	16	830	0.0038	0.021
SkeletAug	GO:0004857	F	enzyme inhibitor activity	6	164	0.0041	0.021
SkeletAug	GO:0017076	F	purine nucleotide binding	61	4945	0.005	0.024
SkeletAug	GO:0003924	F	GTPase activity	5	137	0.0087	0.038
SkeletAug	GO:0005524	F	ATP binding	54	4427	0.01	0.038
SkeletAug	GO:0046983	F	protein dimerization activity	7	259	0.0097	0.038
SkeletAug	GO:0005198	F	structural molecule activity	11	539	0.01	0.038
SkeletAug	GO:0032559	F	adenyl ribonucleotide binding	54	4429	0.011	0.038
SkeletAug	GO:0016887	F	ATPase activity	12	633	0.013	0.044
SkeletAug	GO:0044464	C	cell part	106	7109	1.10E-08	1.40E-07
SkeletAug	GO:0005623	C	cell	106	7109	1.10E-08	1.40E-07
SkeletAug	GO:0016020	C	membrane	50	3219	8.70E-05	0.00073
SkeletAug	GO:0016021	C	integral to membrane	22	1082	0.00036	0.0023
SkeletAug	GO:0044424	C	intracellular part	50	3536	0.00077	0.0035
SkeletAug	GO:0005618	C	cell wall	7	164	0.00085	0.0035
SkeletAug	GO:0005622	C	intracellular	57	4246	0.0011	0.0039
SkeletAug	GO:0031224	C	intrinsic to membrane	23	1270	0.0012	0.0039
SkeletAug	GO:0044425	C	membrane part	26	1551	0.0018	0.005
SkeletAug	GO:0043229	C	intracellular organelle	40	2821	0.0026	0.0054
SkeletAug	GO:0043226	C	organelle	40	2821	0.0026	0.0054
SkeletAug	GO:0032991	C	macromolecular complex	25	1499	0.0023	0.0054
SkeletAug	GO:0030312	C	external encapsulating structure	7	223	0.0045	0.0087
SkeletAug	GO:0043231	C	intracellular membrane-bounded organelle	29	2042	0.0098	0.017
SkeletAug	GO:0043227	C	membrane-bounded organelle	29	2055	0.011	0.018
SkeletAug	GO:0005634	C	nucleus	24	1626	0.012	0.019
SkeletAug	GO:0043234	C	protein complex	16	965	0.014	0.021
SkeletJune	GO:0008152	P	metabolic process	493	12037	2.20E-29	4.10E-27
SkeletJune	GO:0044238	P	primary metabolic process	392	9502	1.90E-21	1.80E-19
SkeletJune	GO:0009987	P	cellular process	405	10572	1.50E-16	9.20E-15
SkeletJune	GO:0044237	P	cellular metabolic process	321	8506	1.90E-11	8.80E-10
SkeletJune	GO:0006139	P	nucleobase, nucleoside, nucleotide and nucleic acid metabolic process	150	3290	3.60E-10	1.40E-08
SkeletJune	GO:0043170	P	macromolecule metabolic process	284	7539	7.40E-10	2.00E-08
SkeletJune	GO:0065007	P	biological regulation	125	2601	7.00E-10	2.00E-08
SkeletJune	GO:0010467	P	gene expression	131	2807	1.50E-09	3.50E-08
SkeletJune	GO:0006807	P	nitrogen compound metabolic process	163	3784	2.80E-09	5.30E-08
SkeletJune	GO:0009058	P	biosynthetic process	179	4271	2.80E-09	5.30E-08
SkeletJune	GO:0009889	P	regulation of biosynthetic process	90	1720	5.50E-09	6.40E-08
SkeletJune	GO:0031326	P	regulation of cellular biosynthetic process	90	1720	5.50E-09	6.40E-08
SkeletJune	GO:0045449	P	regulation of transcription	90	1713	4.60E-09	6.40E-08
SkeletJune	GO:0010556	P	regulation of macromolecule biosynthetic process	90	1720	5.50E-09	6.40E-08

Trait	GO term	Ontology	Description	N in QTL	N in genome	p	FDR
SkeletJune	GO:0010468	P	regulation of gene expression	91	1734	3.90E-09	6.40E-08
SkeletJune	GO:0019219	P	regulation of nucleobase, nucleoside, nucleotide and nucleic acid metabolic process	90	1717	5.10E-09	6.40E-08
SkeletJune	GO:0060255	P	regulation of macromolecule metabolic process	94	1837	6.90E-09	7.20E-08
SkeletJune	GO:0050794	P	regulation of cellular process	112	2336	6.90E-09	7.20E-08
SkeletJune	GO:0080090	P	regulation of primary metabolic process	93	1821	9.20E-09	8.60E-08
SkeletJune	GO:0050789	P	regulation of biological process	117	2490	8.90E-09	8.60E-08
SkeletJune	GO:0031323	P	regulation of cellular metabolic process	90	1743	1.00E-08	8.90E-08
SkeletJune	GO:0051171	P	regulation of nitrogen compound metabolic process	90	1755	1.40E-08	1.10E-07
SkeletJune	GO:0044249	P	cellular biosynthetic process	166	4007	2.70E-08	2.20E-07
SkeletJune	GO:0019222	P	regulation of metabolic process	94	1898	3.10E-08	2.40E-07
SkeletJune	GO:0006350	P	transcription	92	1864	5.00E-08	3.80E-07
SkeletJune	GO:0044260	P	cellular macromolecule metabolic process	248	6824	3.30E-07	2.30E-06
SkeletJune	GO:0005975	P	carbohydrate metabolic process	62	1136	3.90E-07	2.70E-06
SkeletJune	GO:0034645	P	cellular macromolecule biosynthetic process	130	3130	1.00E-06	6.80E-06
SkeletJune	GO:0009059	P	macromolecule biosynthetic process	130	3136	1.10E-06	7.20E-06
SkeletJune	GO:0006629	P	lipid metabolic process	44	731	1.90E-06	1.20E-05
SkeletJune	GO:0016070	P	RNA metabolic process	67	1447	2.50E-05	0.00015
SkeletJune	GO:0006351	P	transcription, DNA-dependent	50	1079	0.00026	0.0015
SkeletJune	GO:0032774	P	RNA biosynthetic process	50	1083	0.00029	0.0016
SkeletJune	GO:0006355	P	regulation of transcription, DNA-dependent	47	1027	0.00051	0.0027
SkeletJune	GO:0006633	P	fatty acid biosynthetic process	10	96	0.00049	0.0027
SkeletJune	GO:0051252	P	regulation of RNA metabolic process	47	1027	0.00051	0.0027
SkeletJune	GO:0051649	P	establishment of localization in cell	20	323	0.00084	0.0043
SkeletJune	GO:0051234	P	establishment of localization	75	1911	0.0011	0.0052
SkeletJune	GO:0006810	P	transport	75	1911	0.0011	0.0052
SkeletJune	GO:0005976	P	polysaccharide metabolic process	14	193	0.0012	0.0058
SkeletJune	GO:0046483	P	heterocycle metabolic process	23	414	0.0014	0.0065
SkeletJune	GO:0051179	P	localization	75	1942	0.0016	0.0073
SkeletJune	GO:0006220	P	pyrimidine nucleotide metabolic process	5	29	0.0018	0.008
SkeletJune	GO:0032787	P	monocarboxylic acid metabolic process	12	159	0.002	0.0084
SkeletJune	GO:0006631	P	fatty acid metabolic process	10	118	0.0021	0.0085
SkeletJune	GO:0009056	P	catabolic process	46	1073	0.0021	0.0085
SkeletJune	GO:0046907	P	intracellular transport	16	255	0.0024	0.0092
SkeletJune	GO:0051641	P	cellular localization	20	354	0.0023	0.0092
SkeletJune	GO:0006886	P	intracellular protein transport	14	212	0.0028	0.011
SkeletJune	GO:0006364	P	rRNA processing	5	33	0.003	0.011
SkeletJune	GO:0016072	P	rRNA metabolic process	5	33	0.003	0.011
SkeletJune	GO:0055086	P	nucleobase, nucleoside and nucleotide metabolic process	19	349	0.0043	0.015
SkeletJune	GO:0009117	P	nucleotide metabolic process	17	298	0.0044	0.015
SkeletJune	GO:0006753	P	nucleoside phosphate metabolic process	17	298	0.0044	0.015
SkeletJune	GO:0006508	P	proteolysis	35	799	0.0049	0.016
SkeletJune	GO:0009057	P	macromolecule catabolic process	41	976	0.0049	0.016
SkeletJune	GO:0044264	P	cellular polysaccharide metabolic process	10	137	0.0056	0.018
SkeletJune	GO:0070727	P	cellular macromolecule localization	14	234	0.0063	0.02
SkeletJune	GO:0034613	P	cellular protein localization	14	234	0.0063	0.02
SkeletJune	GO:0019725	P	cellular homeostasis	12	187	0.0067	0.021
SkeletJune	GO:0042592	P	homeostatic process	12	188	0.0069	0.021
SkeletJune	GO:0065008	P	regulation of biological quality	13	217	0.0082	0.025
SkeletJune	GO:0045454	P	cell redox homeostasis	11	172	0.0094	0.028
SkeletJune	GO:0045184	P	establishment of protein localization	16	303	0.011	0.031
SkeletJune	GO:0015031	P	protein transport	16	303	0.011	0.031
SkeletJune	GO:0034470	P	ncRNA processing	8	108	0.012	0.032

Trait	GO term	Ontology	Description	N in QTL	N in genome	p	FDR
SkeletJune	GO:0019538	P	protein metabolic process	146	4499	0.012	0.032
SkeletJune	GO:0009116	P	nucleoside metabolic process	7	86	0.011	0.032
SkeletJune	GO:0034660	P	ncRNA metabolic process	12	203	0.012	0.032
SkeletJune	GO:0009141	P	nucleoside triphosphate metabolic process	11	185	0.015	0.04
SkeletJune	GO:0042254	P	ribosome biogenesis	5	51	0.016	0.041
SkeletJune	GO:0006820	P	anion transport	6	72	0.017	0.041
SkeletJune	GO:0051716	P	cellular response to stimulus	10	164	0.017	0.041
SkeletJune	GO:0044092	P	negative regulation of molecular function	6	71	0.016	0.041
SkeletJune	GO:0006281	P	DNA repair	10	163	0.017	0.041
SkeletJune	GO:0033554	P	cellular response to stress	10	164	0.017	0.041
SkeletJune	GO:0043086	P	negative regulation of catalytic activity	6	71	0.016	0.041
SkeletJune	GO:0006974	P	response to DNA damage stimulus	10	164	0.017	0.041
SkeletJune	GO:0044255	P	cellular lipid metabolic process	17	345	0.016	0.041
SkeletJune	GO:0022613	P	ribonucleoprotein complex biogenesis	5	53	0.018	0.042
SkeletJune	GO:0033036	P	macromolecule localization	17	353	0.019	0.045
SkeletJune	GO:0042545	P	cell wall modification	7	97	0.02	0.046
SkeletJune	GO:0006073	P	cellular glucan metabolic process	8	122	0.022	0.048
SkeletJune	GO:0006732	P	coenzyme metabolic process	7	99	0.022	0.048
SkeletJune	GO:0044042	P	glucan metabolic process	8	122	0.022	0.048
SkeletJune	GO:0016053	P	organic acid biosynthetic process	13	250	0.023	0.049
SkeletJune	GO:0046394	P	carboxylic acid biosynthetic process	13	250	0.023	0.049
SkeletJune	GO:0003824	F	catalytic activity	504	12409	1.70E-29	2.30E-27
SkeletJune	GO:0016787	F	hydrolase activity	191	3729	1.30E-17	8.50E-16
SkeletJune	GO:0005488	F	binding	450	12383	6.40E-15	2.90E-13
SkeletJune	GO:0043169	F	cation binding	120	2576	9.00E-09	2.40E-07
SkeletJune	GO:0043167	F	ion binding	120	2576	9.00E-09	2.40E-07
SkeletJune	GO:0046914	F	transition metal ion binding	104	2131	1.10E-08	2.40E-07
SkeletJune	GO:0046872	F	metal ion binding	118	2557	2.00E-08	3.90E-07
SkeletJune	GO:0005506	F	iron ion binding	51	780	2.60E-08	4.40E-07
SkeletJune	GO:0016798	F	hydrolase activity, acting on glycosyl bonds	44	623	3.10E-08	4.60E-07
SkeletJune	GO:0004497	F	monooxygenase activity	42	583	3.80E-08	5.10E-07
SkeletJune	GO:0004553	F	hydrolase activity, hydrolyzing O-glycosyl compounds	42	593	5.90E-08	7.30E-07
SkeletJune	GO:0046906	F	tetrapyrrole binding	45	699	2.60E-07	2.90E-06
SkeletJune	GO:0020037	F	heme binding	44	689	4.30E-07	4.50E-06
SkeletJune	GO:0009055	F	electron carrier activity	52	893	5.70E-07	5.20E-06
SkeletJune	GO:0003677	F	DNA binding	93	2000	5.40E-07	5.20E-06
SkeletJune	GO:0016491	F	oxidoreductase activity	105	2435	2.70E-06	2.30E-05
SkeletJune	GO:0004091	F	carboxylesterase activity	20	212	3.90E-06	3.10E-05
SkeletJune	GO:0030528	F	transcription regulator activity	67	1374	5.30E-06	4.00E-05
SkeletJune	GO:0003676	F	nucleic acid binding	139	3583	1.30E-05	8.80E-05
SkeletJune	GO:0004806	F	triglyceride lipase activity	10	59	1.20E-05	8.80E-05
SkeletJune	GO:0004175	F	endopeptidase activity	29	429	1.50E-05	9.50E-05
SkeletJune	GO:0016788	F	hydrolase activity, acting on ester bonds	44	830	3.70E-05	0.00023
SkeletJune	GO:0016298	F	lipase activity	10	69	4.20E-05	0.00024
SkeletJune	GO:0016757	F	transferase activity, transferring glycosyl groups	38	678	4.10E-05	0.00024
SkeletJune	GO:0003700	F	transcription factor activity	43	891	0.00031	0.0017
SkeletJune	GO:0016746	F	transferase activity, transferring acyl groups	23	393	0.00074	0.0039
SkeletJune	GO:0016616	F	oxidoreductase activity, acting on the CH-OH group of donors, NAD or NADP as accepto	13	167	0.001	0.0051
SkeletJune	GO:0008233	F	peptidase activity	34	699	0.0011	0.0055
SkeletJune	GO:0070011	F	peptidase activity, acting on L-amino acid peptides	33	676	0.0013	0.0059
SkeletJune	GO:0004252	F	serine-type endopeptidase activity	12	162	0.0023	0.0088
SkeletJune	GO:0051287	F	NAD or NADH binding	8	80	0.0022	0.0088

Trait	GO term	Ontology	Description	N in QTL	N in genome	p	FDR
SkeletJune	GO:0016818	F	hydrolase activity, acting on acid anhydrides, in phosphorus-containing anhydrides	46	1077	0.0022	0.0088
SkeletJune	GO:0016758	F	transferase activity, transferring hexosyl groups	28	562	0.0022	0.0088
SkeletJune	GO:0042623	F	ATPase activity, coupled	20	352	0.0022	0.0088
SkeletJune	GO:0004222	F	metalloendopeptidase activity	8	79	0.0021	0.0088
SkeletJune	GO:0008170	F	N-methyltransferase activity	5	31	0.0024	0.009
SkeletJune	GO:0016614	F	oxidoreductase activity, acting on CH-OH group of donors	13	192	0.0032	0.011
SkeletJune	GO:0043565	F	sequence-specific DNA binding	29	606	0.0031	0.011
SkeletJune	GO:0008270	F	zinc ion binding	48	1165	0.0035	0.012
SkeletJune	GO:0016740	F	transferase activity	158	4741	0.0034	0.012
SkeletJune	GO:0008415	F	acyltransferase activity	14	218	0.0035	0.012
SkeletJune	GO:0000166	F	nucleotide binding	168	5094	0.0037	0.012
SkeletJune	GO:0032555	F	purine ribonucleotide binding	156	4698	0.0041	0.012
SkeletJune	GO:0032553	F	ribonucleotide binding	156	4698	0.0041	0.012
SkeletJune	GO:0015035	F	protein disulfide oxidoreductase activity	7	70	0.0041	0.012
SkeletJune	GO:0015036	F	disulfide oxidoreductase activity	7	70	0.0041	0.012
SkeletJune	GO:0004386	F	helicase activity	16	276	0.0049	0.014
SkeletJune	GO:0008509	F	anion transmembrane transporter activity	6	55	0.0053	0.015
SkeletJune	GO:0030599	F	pectinesterase activity	10	136	0.0054	0.015
SkeletJune	GO:0016817	F	hydrolase activity, acting on acid anhydrides	46	1136	0.0056	0.015
SkeletJune	GO:0016462	F	pyrophosphatase activity	43	1052	0.0063	0.017
SkeletJune	GO:0017111	F	nucleoside-triphosphatase activity	42	1030	0.0071	0.018
SkeletJune	GO:0005524	F	ATP binding	146	4427	0.0069	0.018
SkeletJune	GO:0032559	F	adenyl ribonucleotide binding	146	4429	0.007	0.018
SkeletJune	GO:0016747	F	transferase activity, transferring acyl groups other than amino-acyl groups	19	371	0.0079	0.02
SkeletJune	GO:0043492	F	ATPase activity, coupled to movement of substances	11	177	0.011	0.027
SkeletJune	GO:0042626	F	ATPase activity, coupled to transmembrane movement of substances	11	177	0.011	0.027
SkeletJune	GO:0008236	F	serine-type peptidase activity	15	282	0.013	0.029
SkeletJune	GO:0017171	F	serine hydrolase activity	15	282	0.013	0.029
SkeletJune	GO:0030234	F	enzyme regulator activity	16	311	0.013	0.031
SkeletJune	GO:0022892	F	substrate-specific transporter activity	29	683	0.014	0.032
SkeletJune	GO:0016667	F	oxidoreductase activity, acting on sulfur group of donors	7	92	0.016	0.034
SkeletJune	GO:0016887	F	ATPase activity	27	633	0.017	0.036
SkeletJune	GO:0015075	F	ion transmembrane transporter activity	24	546	0.017	0.036
SkeletJune	GO:0004857	F	enzyme inhibitor activity	10	164	0.017	0.036
SkeletJune	GO:0017076	F	purine nucleotide binding	157	4945	0.018	0.037
SkeletJune	GO:0008237	F	metallopeptidase activity	8	121	0.021	0.042
SkeletJune	GO:0022804	F	active transmembrane transporter activity	19	412	0.021	0.042
SkeletJune	GO:0022857	F	transmembrane transporter activity	34	861	0.022	0.042
SkeletJune	GO:0022891	F	substrate-specific transmembrane transporter activity	25	595	0.024	0.047
SkeletJune	GO:0042802	F	identical protein binding	6	80	0.026	0.049
SkeletJune	GO:0030554	F	adenyl nucleotide binding	147	4664	0.027	0.05
SkeletJune	GO:0022836	F	gated channel activity	7	104	0.027	0.05
SkeletJune	GO:0001883	F	purine nucleoside binding	147	4664	0.027	0.05
SkeletJune	GO:0001882	F	nucleoside binding	147	4666	0.028	0.05
SkeletJune	GO:0044464	C	cell part	293	7109	9.00E-15	1.40E-13
SkeletJune	GO:0005622	C	intracellular	200	4246	7.20E-15	1.40E-13
SkeletJune	GO:0005623	C	cell	293	7109	9.00E-15	1.40E-13
SkeletJune	GO:0044424	C	intracellular part	172	3536	6.90E-14	8.30E-13
SkeletJune	GO:0043231	C	intracellular membrane-bounded organelle	110	2042	1.80E-11	1.50E-10
SkeletJune	GO:0043229	C	intracellular organelle	138	2821	2.40E-11	1.50E-10
SkeletJune	GO:0043227	C	membrane-bounded organelle	110	2055	2.50E-11	1.50E-10
SkeletJune	GO:0043226	C	organelle	138	2821	2.40E-11	1.50E-10

Trait	GO term	Ontology	Description	N in QTL	N in genome	p	FDR
SkeletJune	GO:0005634	C	nucleus	81	1626	2.40E-07	1.30E-06
SkeletJune	GO:0005737	C	cytoplasm	71	1393	6.20E-07	3.00E-06
SkeletJune	GO:0005783	C	endoplasmic reticulum	16	153	1.10E-05	4.80E-05
SkeletJune	GO:0044444	C	cytoplasmic part	53	1085	4.90E-05	0.0002
SkeletJune	GO:0044422	C	organelle part	36	680	0.00019	0.00064
SkeletJune	GO:0044446	C	intracellular organelle part	36	680	0.00019	0.00064
SkeletJune	GO:0031090	C	organelle membrane	16	246	0.0017	0.0054
SkeletJune	GO:0016020	C	membrane	114	3219	0.0021	0.006
SkeletJune	GO:0031975	C	envelope	14	204	0.002	0.006
SkeletJune	GO:0012505	C	endomembrane system	13	184	0.0023	0.006
SkeletJune	GO:0031967	C	organelle envelope	11	145	0.0029	0.0073
SkeletJune	GO:0019867	C	outer membrane	5	34	0.0034	0.0082
SkeletJune	GO:0030312	C	external encapsulating structure	14	223	0.0043	0.0097
SkeletJune	GO:0005618	C	cell wall	11	164	0.0068	0.015
SkeletJune	GO:0005643	C	nuclear pore	5	49	0.014	0.026
SkeletJune	GO:0005635	C	nuclear envelope	5	49	0.014	0.026
SkeletJune	GO:0046930	C	pore complex	5	49	0.014	0.026
SkeletJune	GO:0031966	C	mitochondrial membrane	6	74	0.019	0.035
SkeletJune	GO:0016021	C	integral to membrane	41	1082	0.022	0.039
SkeletJune	GO:0032991	C	macromolecular complex	54	1499	0.023	0.039
SkeletJune	GO:0005576	C	extracellular region	6	81	0.027	0.045
