# Supplementary material for: Insight into the Genetic Components of Community Genetics: QTL Mapping of Insect Association in a Fast-Growing Forest Tree
Source: PLoS One. 2013 Nov 19;8(11):e79925. doi: 10.1371/journal.pone.0079925 (PMC3833894; doi:10.1371/journal.pone.0079925)

June miner

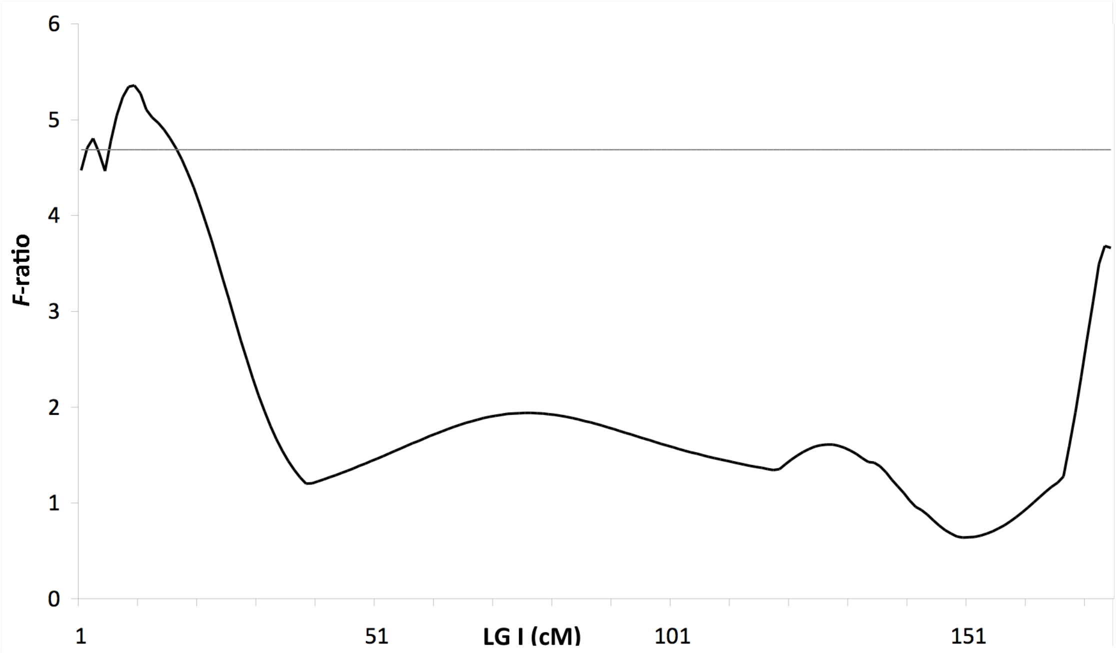

June chewer

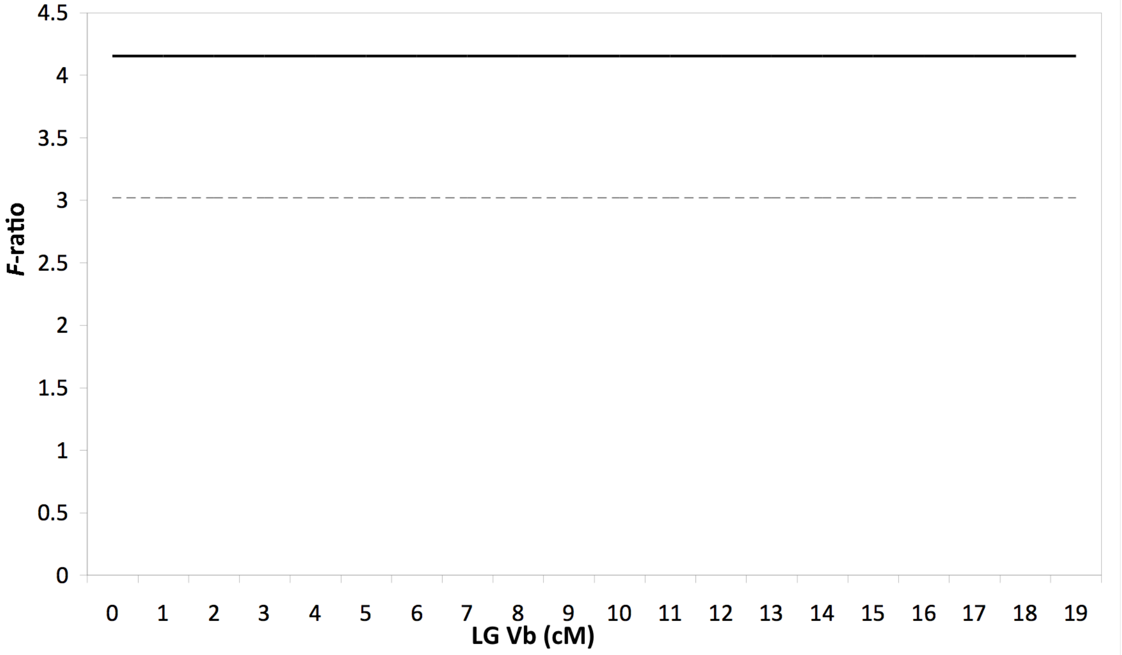

June skeletonizer

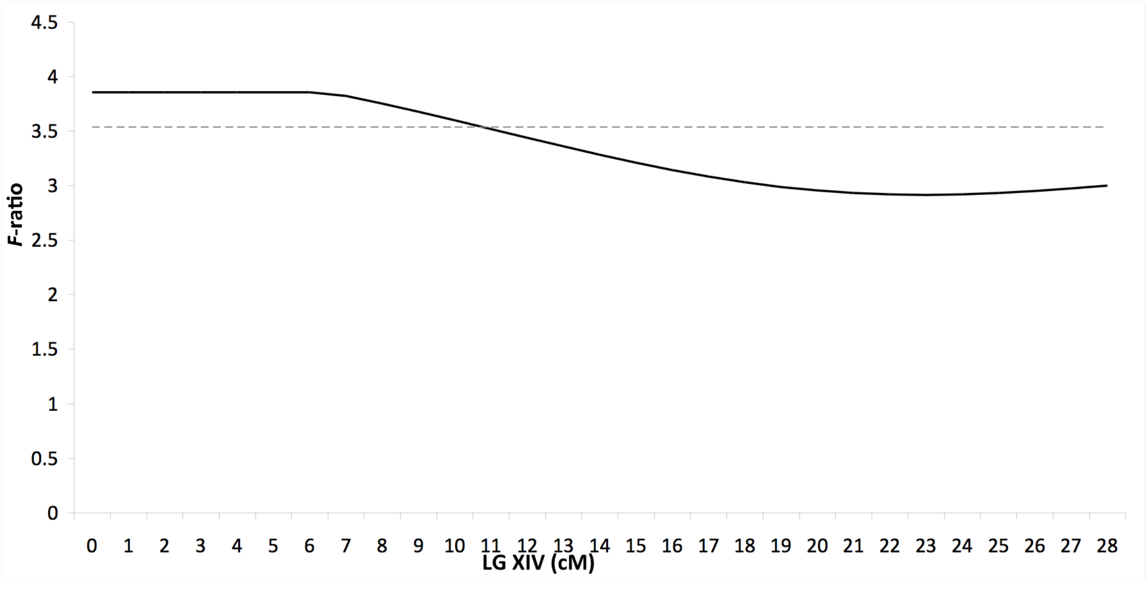

August sap suckers (leaves)

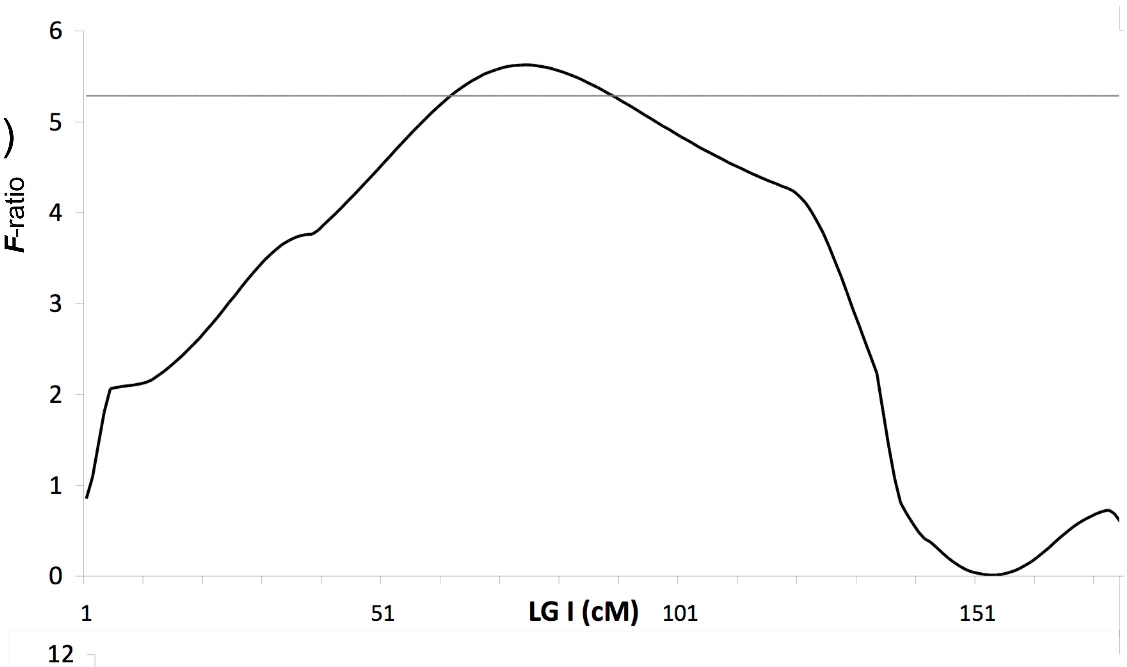

August chewers

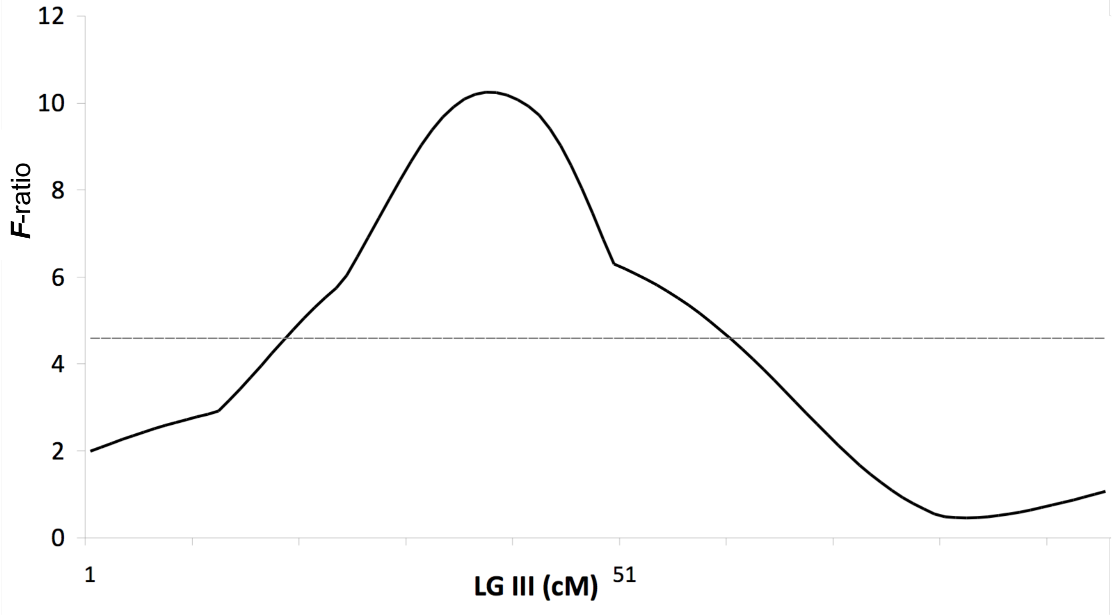

August skeletonizers

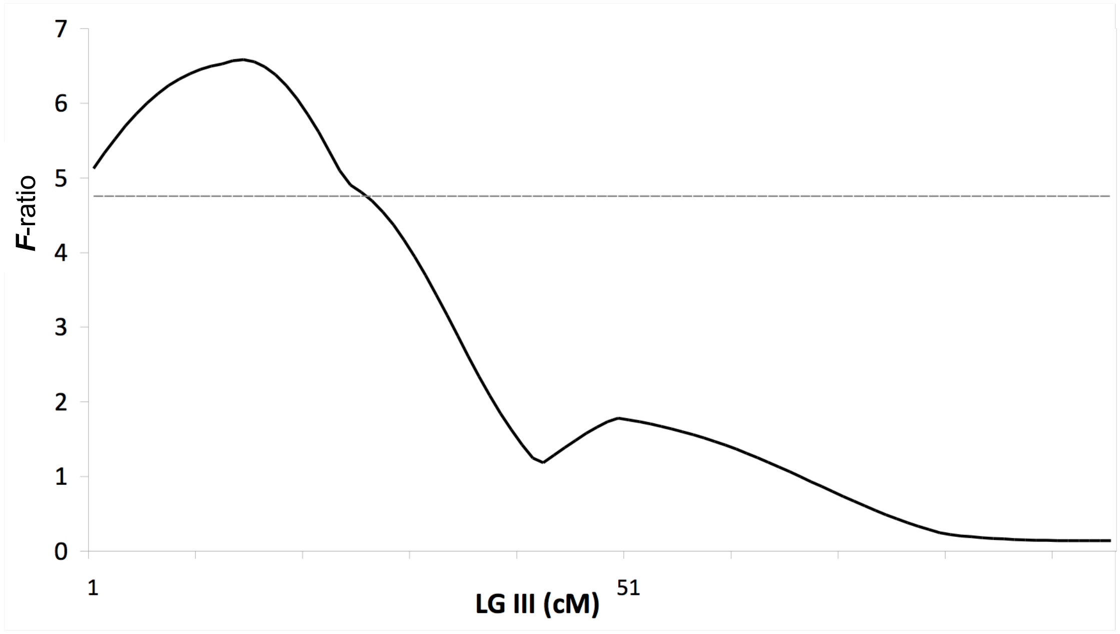

August chewers

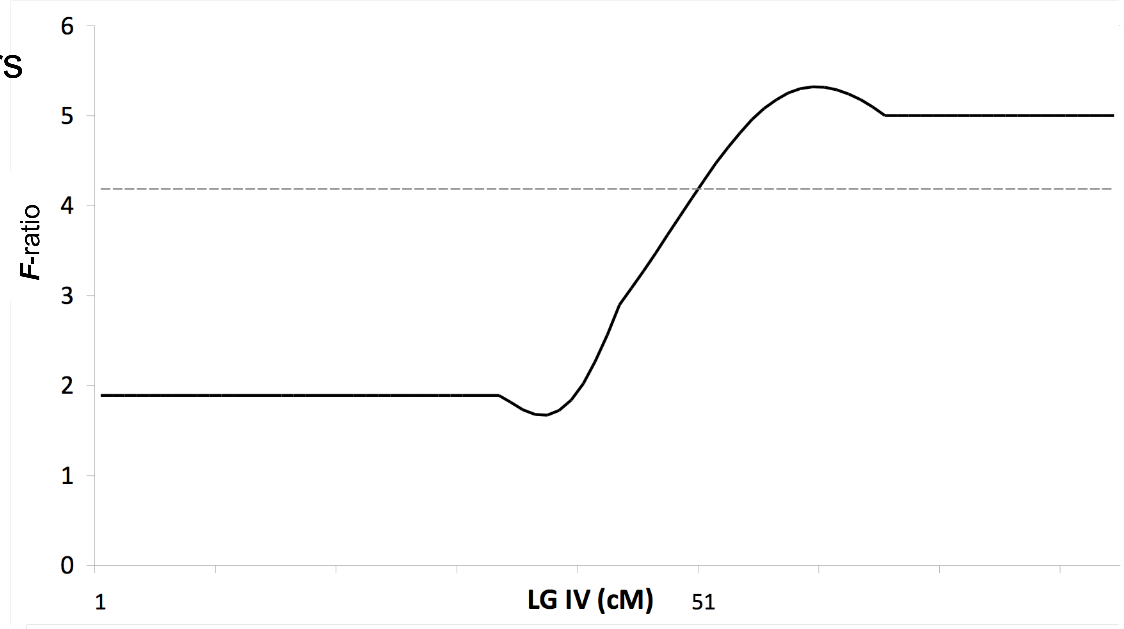

August chewers

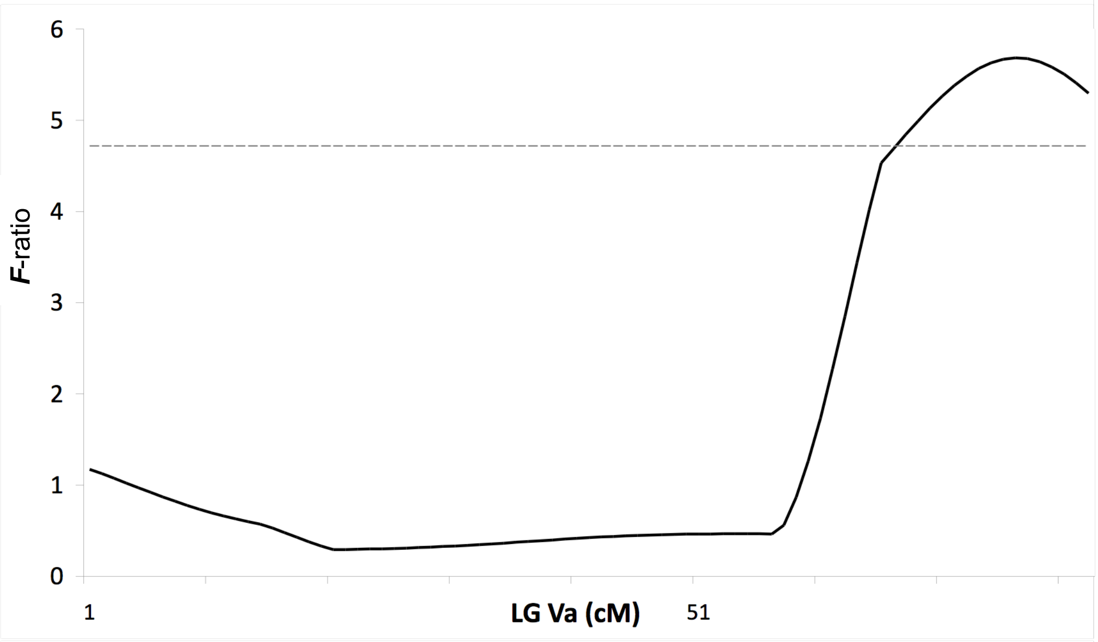

August miners

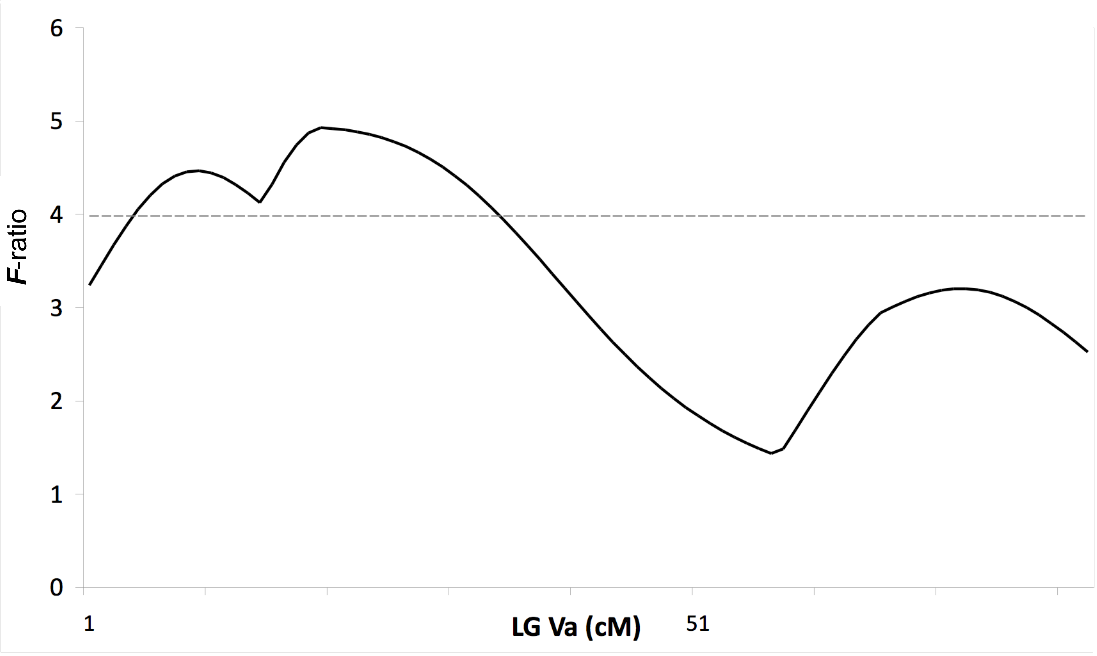

August sap suckers (leaves)

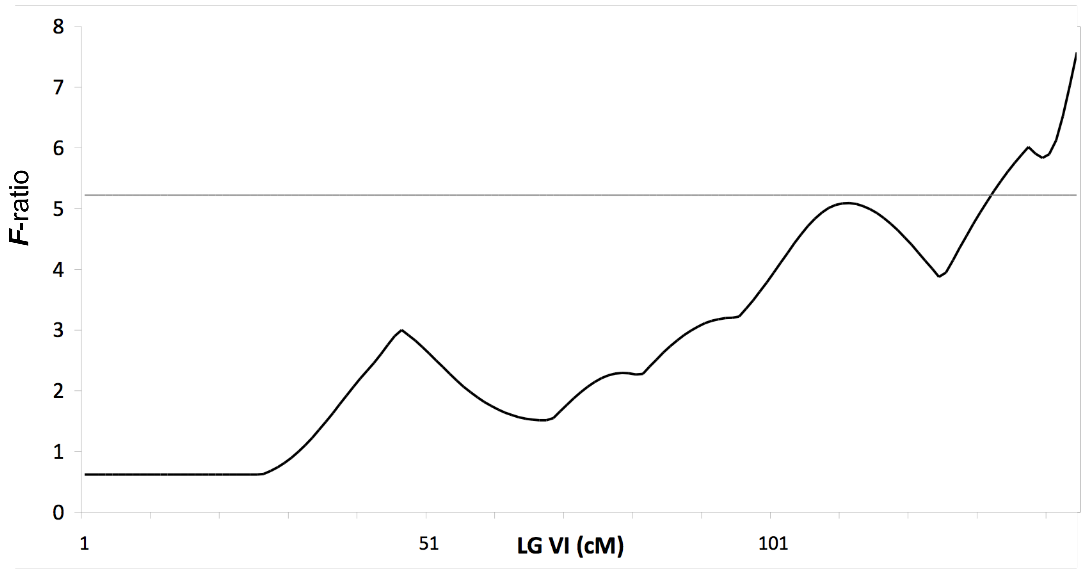

August miners

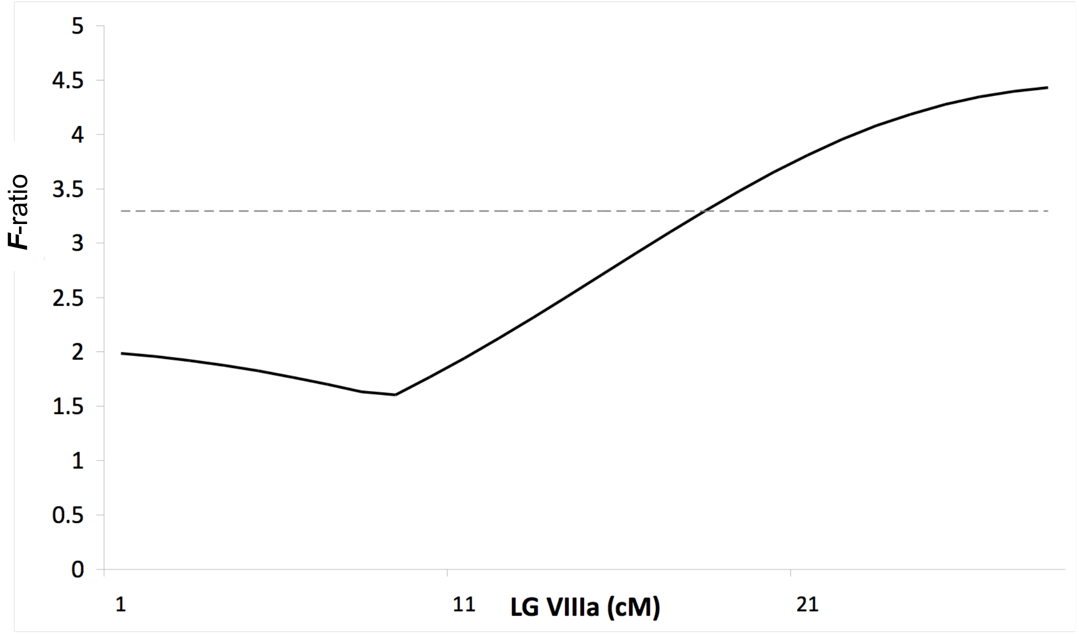

August sap suckers (leaves)

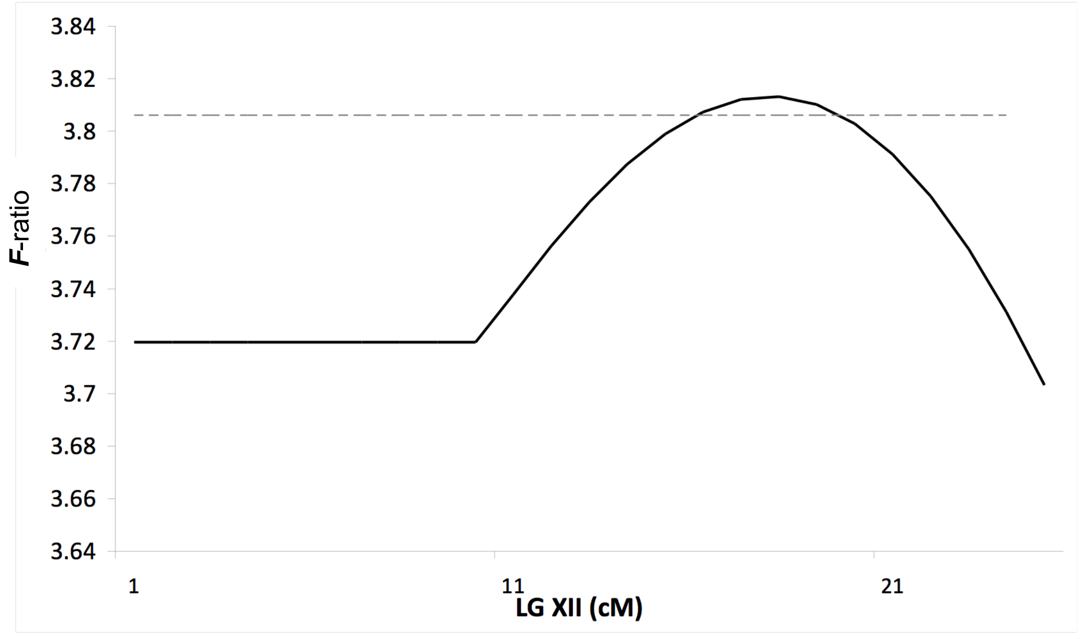

August chewers

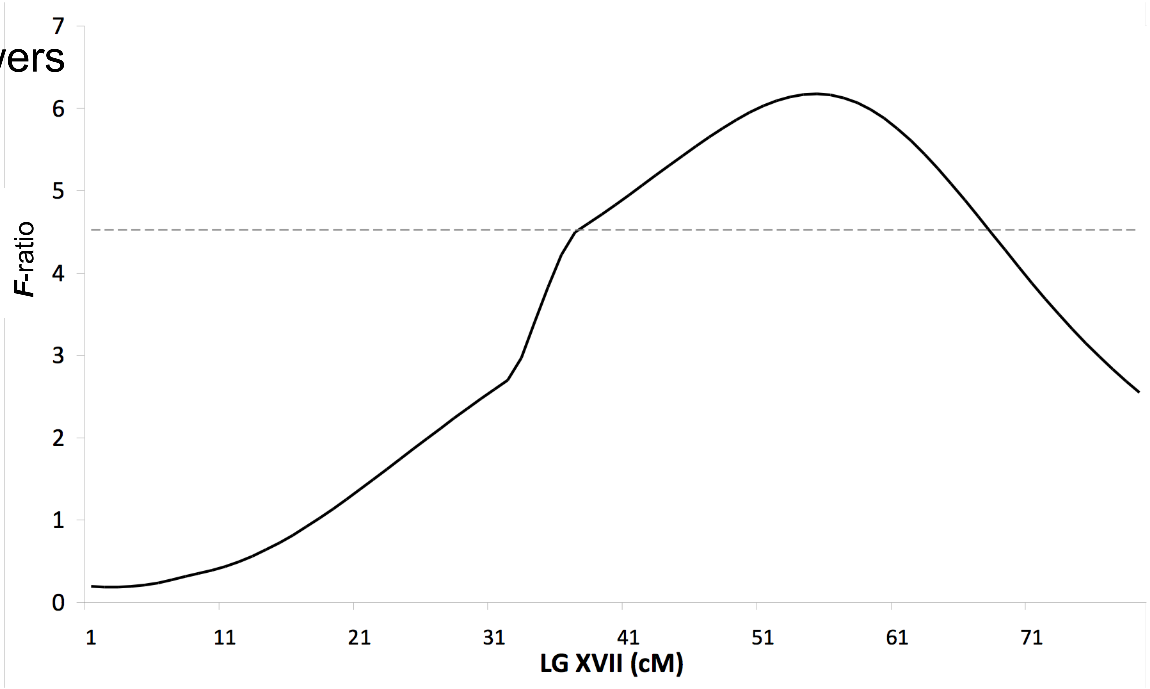

August miners

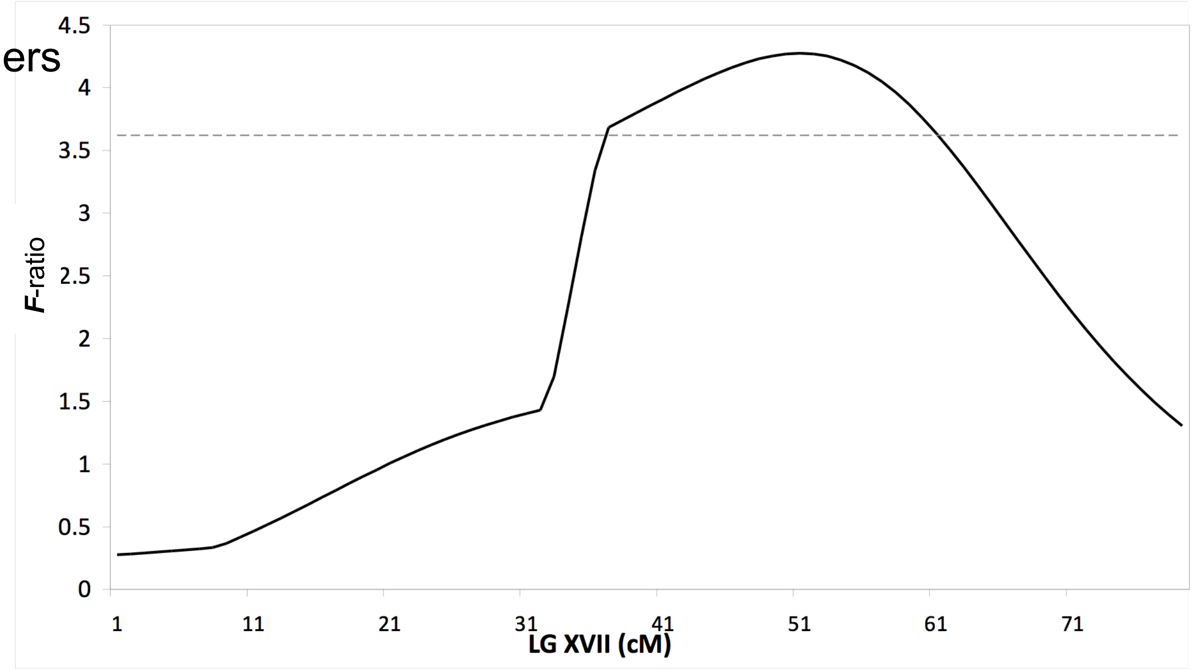

Supplement: Figure S1 — Test statistics across each chromosome, calculated as F - ratios, were used to identify significant QTL for each month/damage category. Each horizontal axis represents the length of one linkage group from the F2 linkage map. The vertical axis indicates the magnitude of the test statistic. Solid lines represent the observed F-ratio, and dashed lines represent the 5% critical value calculated from 1000 chromosome-wide permutations. (PDF) [file pone.0079925.s004.pdf]
