# Supplementary figures and images for: Insight into the Genetic Components of Community Genetics: QTL Mapping of Insect Association in a Fast-Growing Forest Tree
Source: PLoS One. 2013 Nov 19;8(11):e79925. doi: 10.1371/journal.pone.0079925 (PMC3833894; doi:10.1371/journal.pone.0079925)

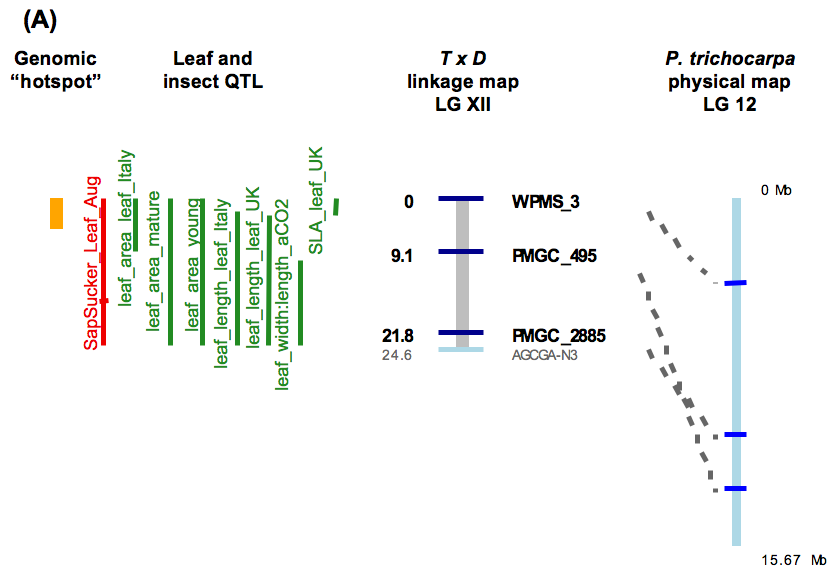

Supplement: Figure S2 — QTL for insect damage (red) co-locate to QTL hotspots (gold) for leaf traits (green) on two linkage groups in a hybrid poplar pedigree ( P. trichocarpa x P. deltoides , T x D ). (A) The presence of sap suckers (primarily aphids) on leaves co-locates with QTL for leaf size on LG XII. (B) The frequency of skeletonizers early in the summer co-locates with QTL for leaf size and growth rate on LG XIV. (TIFF) [file pone.0079925.s005.tif]
